# Extracellular CIRP Dysregulates Microglial Efferocytosis in Acute Ischemic Stroke via the TLR4/miR‐155/MafB Axis

**DOI:** 10.1002/advs.77053

**Published:** 2026-08-15

**Authors:** Dmitriy Lapin, Dilara Aylar, Archna Sharma, Ping Wang

**Affiliations:** ^1^ Center For Immunology and Inflammation The Feinstein Institutes For Medical Research Manhasset New York USA; ^2^ Department of Molecular Medicine Zucker School of Medicine at Hofstra/Northwell Manhasset New York USA; ^3^ Department of Surgery Zucker School of Medicine at Hofstra/Northwell Manhasset New York USA

**Keywords:** C23, eCIRP, efferocytosis, ischemic stroke, microglia, neuroinflammation, TLR4/miR‐155/MafB axis

## Abstract

Efferocytosis, the phagocytic clearance of dying cells, by microglia is crucial for limiting neuroinflammation and promoting resolution in ischemic stroke. Extracellular cold‐inducible RNA‐binding protein (eCIRP) is an inflammatory mediator that impairs macrophage bacterial phagocytosis in sepsis and radiation injury, but its role in microglial efferocytosis in ischemic stroke has not yet been studied. Using a transient middle cerebral artery occlusion (tMCAO) model of ischemic stroke, this study demonstrated that eCIRP is released into the cerebrospinal fluid and microglial expression of the crucial efferocytic receptor MerTK decreases in tMCAO mice. CIRP deficiency significantly improved MerTK expression and microglial efferocytosis in tMCAO mice, reducing brain infarction, inflammation, neurological deficit, and survival in acute stroke. eCIRP induces pro‐inflammatory micro‐RNA 155 (miR‐155) via TLR4, which suppresses its target pro‐efferocytic transcription factor MAF bZIP (MafB), downregulating MerTK and the downstream cytoskeletal regulators, to impair microglial efferocytosis. Pharmacological blockade of eCIRP–TLR4 interaction using small peptide C23 attenuates miR‐155 induction, restores MerTK expression, rescues microglial efferocytosis, and improves outcomes in tMCAO mice. This study uncovers a previously unknown pathway through which eCIRP signaling impairs neuroprotective efferocytic microglial function in ischemic stroke, suggesting that targeting eCIRP may promote functional recovery after stroke.

## Graphical abstract

eCIRP released in the stroke brain binds to TLR4 expressed on microglia to induce miR‐155, which suppresses MafB, decreases MerTK and downstream signaling to impair efferocytosis of apoptotic neurons, worsening the acute stroke outcomes. Inhibition of eCIRP‐TLR4 interaction with small peptide C23 attenuates eCIRP‐induced microglial efferocytic dysfunction and improves stroke outcomes, highlighting its potential to promote functional recovery after stroke.

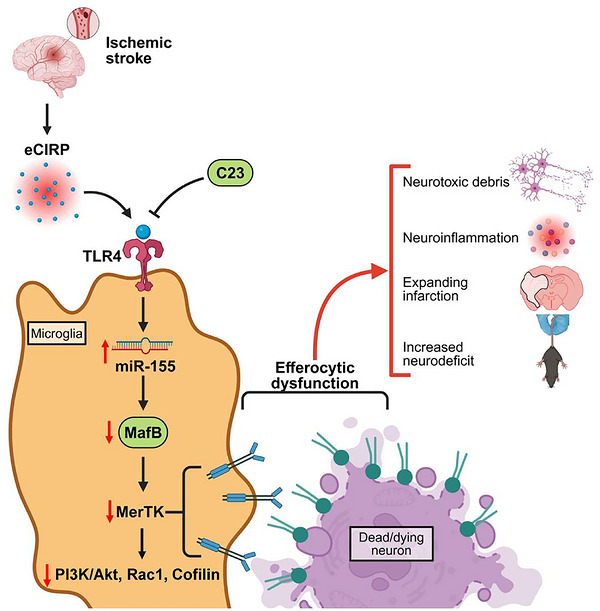

## 1 Introduction

Stroke is the second leading cause of death, and third leading cause of combined death and disability worldwide [1, 2]. Of all stroke subtypes, ischemic stroke (IS) is the most common, making up 65% of all incident strokes [2]. Despite the massive global burden of IS, there has been a distinct lack of success in the development of novel efficacious therapies for IS [3]. During and after cessation of ischemia, brain cells release soluble pro‐inflammatory molecules such as cytokines and damage‐associated molecular patterns (DAMPs), producing a pro‐inflammatory milieu [4]. Although the post‐stroke inflammatory response is necessary for eventual tissue repair, the exaggerated acute phase of this response is known to amplify tissue damage and expand infarction, representing an opportunity for targeted pharmacologic intervention [5].

Cold‐inducible RNA‐binding protein (CIRP) is a small protein that is ubiquitously and constitutively expressed, canonically known to function as an RNA chaperone [6, 7]. We discovered that CIRP can be released extracellularly into circulation in sepsis and hemorrhagic shock [8]. Extracellular CIRP (eCIRP) then acts as a pro‐inflammatory DAMP by binding to pattern recognition receptors toll‐like receptor 4 (TLR4), triggering receptor expressed on myeloid cells‐1 (TREM‐1), and interleukin 6 receptor α (IL‐6Rα) to induce deleterious inflammatory signaling pathways [8, 9, 10, 11]. Recent studies in IS have demonstrated eCIRP's injurious role, underscoring the need to improve our understanding of eCIRP dynamics in IS [12, 13, 14, 15, 16]. CIRP deficient (CIRP^−/−^) mice subjected to permanent middle cerebral artery occlusion (pMCAO) exhibit reduced microglial activation and smaller infarcts compared to control mice [12]. A recent study in a murine temporary middle cerebral artery occlusion (tMCAO) model of IS, showed that CIRP expression drives brain expression of pyroptosis‐related proteins, augments pro‐inflammatory polarization in microglia, and increases infarct volume, potentially via the NF‐κB/NLRP3 pathway [15]. eCIRP also disrupts BBB integrity and promotes cerebral edema, a potentially deadly sequela of IS [15]. Human data corroborates eCIRP's role as an important DAMP in IS. Serum eCIRP levels in patients correlate with clinical IS severity and outcomes metrics, including infarct volume, cerebral edema, NIH stroke score, and modified Rankin score [14, 17].

Microglia play pivotal roles in IS as the quintessential CNS resident immune cells. Microglia are a heterogeneous population, exhibiting varying functionality along spatial and temporal dimensions [18]. They can promote or suppress inflammation via multiple mechanisms but have been shown to be beneficial as a whole [19]. Efferocytosis, the phagocytosis of dead and dying cells, is well characterized as beneficial, as loss of function results in worse outcomes [20, 21, 22]. Mer tyrosine kinase (MerTK) is a phosphatidylserine recognition receptor essential for efferocytosis in microglia [23, 24, 25, 26]. One key transcription factor that controls anti‐inflammatory function in microglia and macrophages is MAF bZIP transcription factor B (MafB), a musculoaponeurotic fibrosarcoma family member [27, 28]. MafB has been shown to reduce infarct volume and neuroinflammation in tMCAO by promoting DAMP clearance in peripheral macrophages [29]. Recent transcriptomic data also revealed that MafB is crucial for late‐phase tissue repair function of monocyte‐derived macrophages, including myelin phagocytosis, in ischemic stroke after attenuation of early‐phase microglia [30]. MafB‐high microglia have been associated with brain repair and clears myelin debris [31]. MafB expression is negatively regulated by micro‐RNA 155 (miR‐155), a pro‐inflammatory miRNA that worsens IS in rodents [32, 33]. This inverse relationship between deleterious miR‐155 and protective MafB is supported by data from human IS patient serum, where miR‐155 positively correlates with poor outcomes and inversely correlates with MafB mRNA levels [32, 34, 35].

We recently discovered that eCIRP causes dysfunctional bacterial phagocytosis in peripheral macrophages in the setting of sepsis [36]. Since, much of the cellular machinery of efferocytosis is shared with phagocytosis [37], we hypothesized that eCIRP released in IS, causes microglial efferocytic dysfunction and promotes brain tissue injury. We found that eCIRP is released into the cerebrospinal fluid (CSF) of tMCAO mice and that expression of efferocytic receptor MerTK is decreased in the microglia in tMCAO brains in the acute phase of stroke. CIRP deficiency restored microglial MerTK expression and enhanced microglial efferocytosis of apoptotic neurons in the peri‐infarct brain, improving outcomes in brain infarction, inflammation, neurological function, and overall survival in acute stroke. As seen in WT tMCAO mice, hippocampal injection of eCIRP increased miR‐155 and decreased MafB levels. In contrast, CIRP^−/−^ tMCAO mice had attenuated miR‐155, but preserved MafB expression in the brain compared to WT tMCAO. Next, we employed in vitro primary microglial cell culture to unveil the underlying mechanism. Here we report that eCIRP initiates an efferocytosis‐suppressing signaling pathway via TLR4, inducing pro‐inflammatory miR‐155 and downregulating MafB, thus reducing the expression of pro‐efferocytic MerTK and downstream cytoskeletal rearrangement mediators. Finally, we examined a therapeutic approach to target eCIRP using a small inhibitory peptide C23 to augment efferocytosis and improve outcomes in IS.

## 2 Results

### 2.1 ECIRP is Released into the CSF and Impairs Microglial Efferocytosis in Ischemic Stroke

**FIGURE 1:**
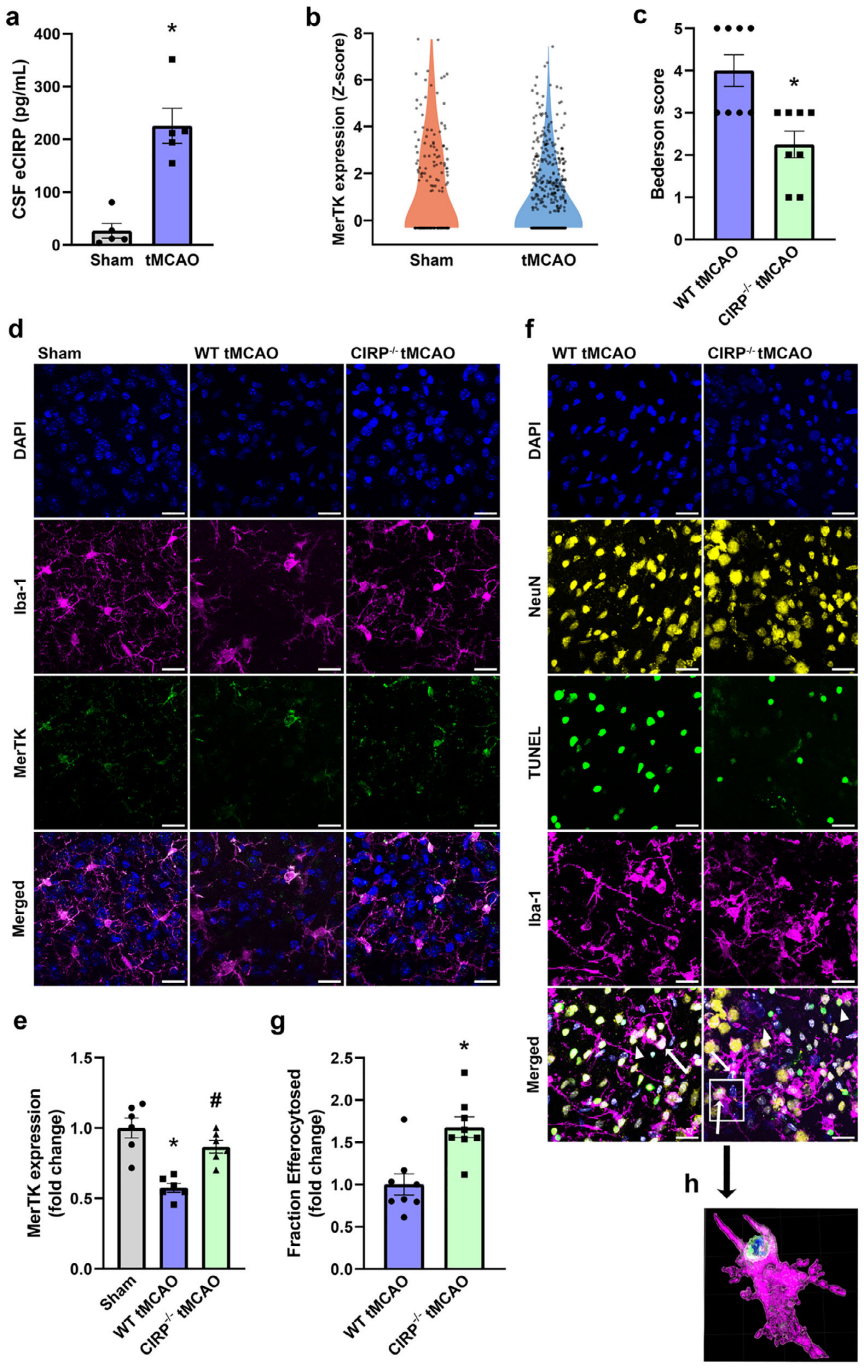
eCIRP is released in ischemic stroke and causes efferocytic dysfunction in vivo. (a) Concentration of eCIRP in CSF collected from mice 24 h after sham or tMCAO surgery. (*n* = 5/group; mean ± SEM; ^*^ *p* = 0.0006 versus Sham; Unpaired *t*‐test). (b) *Mertk* expression Z‐score in microglia cluster from GSE227651. (c) Bederson score in WT tMCAO and CIRP^−/−^ tMCAO mice 24 h after surgery. (*n* = 8/group; mean ± SEM; ^*^ *p* = 0.0031; Unpaired *t*‐test). (d) Representative confocal images of peri‐infarct brain tissues in Sham, WT tMCAO, and CIRP^−/−^ tMCAO mice, stained for DAPI (blue), Iba‐1 (magenta), and MerTK (green). Magnification 630x, scale bar, 20 µM. (e) Quantification of MerTK expression in microglia per animal. (*n* = 6/group; mean ± SEM; ^*^ *p* < 0.0001 versus Sham; ^#^ *p* = 0.0032 versus WT tMCAO; one‐way ANOVA, Tukey's multiple comparisons test). (f) Representative confocal images of peri‐infarct brain tissues in WT tMCAO and CIRP^−/−^ tMCAO mice, with staining for nuclei (DAPI, blue), neurons (NeuN, yellow), dead/dying cells (TUNEL, green), and microglia (Iba‐1, magenta); arrows indicate efferocytosis events with complete internalization of TUNEL^+^/NeuN^+^ cargo, arrowheads indicate incomplete efferocytosis events. Magnification 630x, scale bar, 20 µM. (g) Ratio of TUNEL^+^ neurons efferocytosed by microglia to total TUNEL^+^ neurons expressed as fold‐change with WT tMCAO set as 1. (*n* = 8/group; mean ± SEM; ^*^ *p* = 0.0017 versus WT tMCAO; Unpaired *t*‐test). (h) Zoomed in high power reconstruction of 3D segmented surfaces in Imaris showing efferocytosis event indicated in white box in (f), demonstrating TUNEL^+^/NeuN^+^ cargo engulfed by Iba‐1^+^ microglia.

Despite eCIRP's deleterious role in stroke [12, 13, 14], no direct measurements of eCIRP in the extracellular compartment of the brain, such as CSF, have been published to date. Therefore, to evaluate CSF eCIRP levels in ischemic stroke, we collected CSF from sham and tMCAO mice 24 h post‐operatively and quantified CIRP via ELISA. We found eCIRP levels to be significantly elevated by 8.4‐fold in the CSF of tMCAO mice compared to sham controls (Figure 1a). To confirm that microglial efferocytosis is inefficient in early IS, we interrogated a publicly available single‐cell RNA sequencing data set (GSE227651), generated from sham and tMCAO mice brains, for microglial expression of the efferocytic receptor MerTK [38]. Our analysis revealed that MerTk expression was reduced in microglia 24 h after tMCAO (Figure 1b). We asked whether eCIRP could contribute to early microglial efferocytic dysfunction in IS, and to that end, we subjected C57BL/6 (WT) and CIRP^−/−^ mice to tMCAO. Building on our previous study, which showed that CIRP^−/−^ mice develop smaller infarcts in pMCAO [12], we assessed Bederson score, a measurement of neurological deficit, 24 h after tMCAO surgery to evaluate the IS outcome. As expected, Bederson scores in CIRP^−/−^ tMCAO mice were significantly decreased by 43.8% versus WT counterparts (Figure 1c). Next, we measured microglial MerTK expression in WT and CIRP^−/−^ tMCAO versus sham brains via immunofluorescence co‐staining of Iba‐1 and MerTK (Figure 1d). We screened orthogonal z‐stack views for each image to confirm the colocalization of MerTK with Iba stained microglia (representative image in Figure S1). The MerTK expression of microglia was significantly decreased by 42.6% in WT tMCAO compared to sham control brain tissue (Figure 1d,e). CIRP deficiency rescued the decrease in microglial MerTK expression in tMCAO mice, which was comparable to sham in CIRP^−/−^ tMCAO brain tissue (Figure 1d,e). Finally, we examined microglial efferocytosis of dead/dying neurons in vivo in tMCAO mice, via immunofluorescence staining of brain tissue sections for neuronal marker NeuN and microglial marker Iba‐1, followed by TUNEL labeling to stain dead/dying cells and DAPI staining. Images were acquired from cortical peri‐infarct regions, and efferocytosis was identified as engulfment and internalization of TUNEL^+^/NeuN^+^ cargo by Iba‐1^+^ microglia (Figure 1f,h). Efferocytic events were then quantified utilizing Imaris 3D segmentation software (Figure 1h), which showed that microglial efferocytosis of TUNEL^+^ neurons was significantly increased by 1.7‐fold in CIRP^−/−^ tMCAO brains compared to WT tMCAO brains (Figure 1g).

**FIGURE 2:**
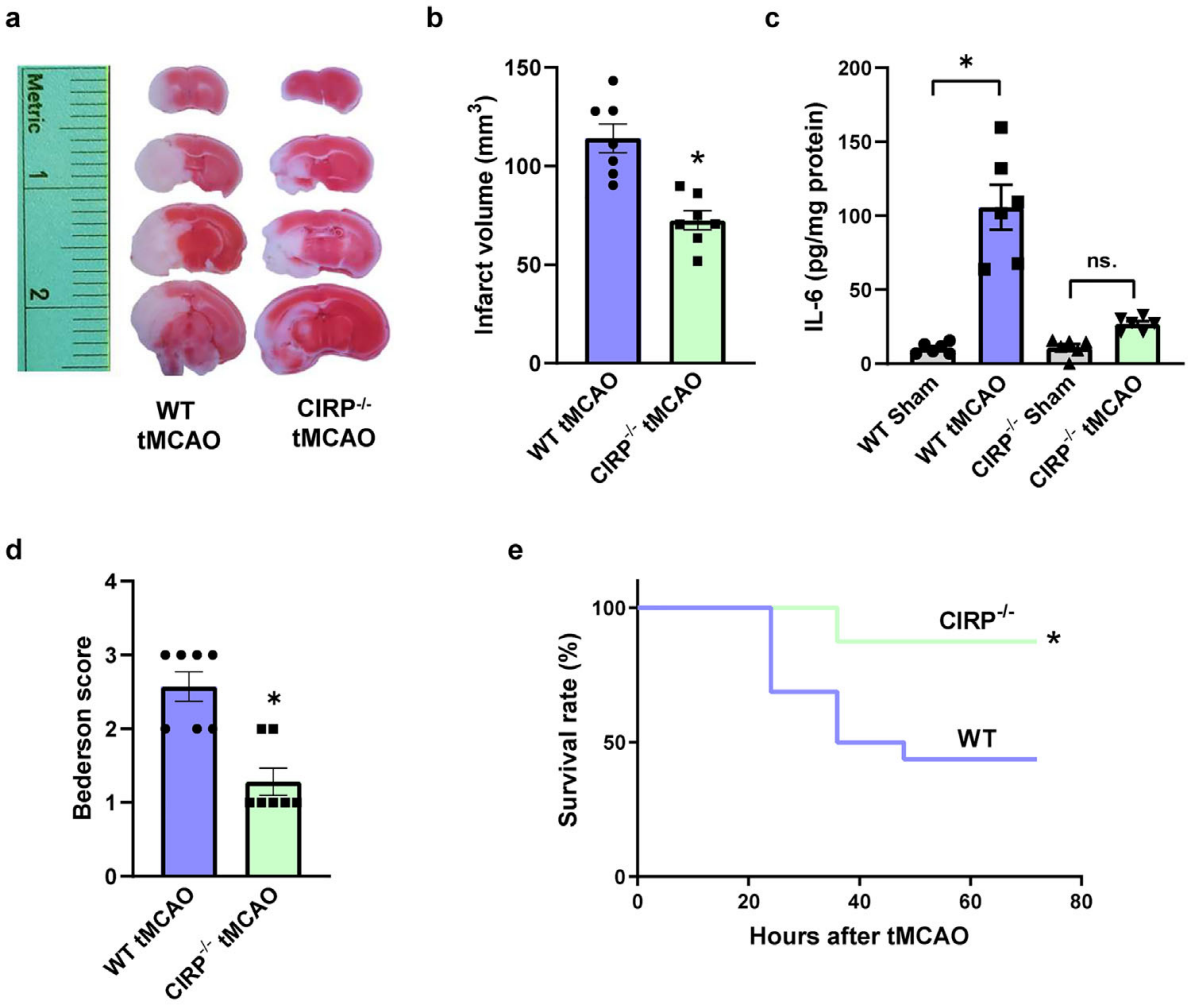
CIRP‐deficiency improves outcomes in acute ischemic stroke. WT and CIRP^−/−^ mice were subjected to Sham or tMCAO surgery, and outcomes assessed 3‐day post‐tMCAO. (a) Representative images of 2 mm thick brain sections stained with TTC for infarction detection and quantification 3‐day post‐tMCAO. Infarcted brain tissue is stained pale white, while healthy brain tissue is stained in red. (b) Infarct volume in WT and CIRP^−/−^ mice 3 days after tMCAO quantified with NIH ImageJ. (*n* = 7/group; mean ± SEM; ^*^ *p* = 0.0005; Unpaired *t*‐test). (c) IL‐6 levels in 3‐day post‐tMCAO total brain lysates expressed as IL‐6 pg/mg total protein. (*n* = 6/group; ^*^ *p* < 0.0001 WT sham versus WT tMCAO; ns. *p* = 0.2885 CIRP^−/−^ sham versus CIRP^−/−^ tMCAO; Mixed‐effects model (REML) with Sidak's multiple comparisons test). (d) Bederson score 3‐day post tMCAO in WT and CIRP^−/−^ mice (*n* = 7/group; mean ± SEM; ^*^ *p* = 0.0005; Unpaired *t*‐test). (e) WT and CIRP^−/−^ mice subjected to tMCAO‐induced ischemic stroke were monitored for 3 days for humane endpoints, and differences in survival were determined using Kaplan‐Meier survival plots and a log‐rank test. *n*  =  8‐16 mice/group. Hazard ratios (HR): WT, 5.557; CIRP^−/−^, 0.1799. *p = 0.0452 versus WT.

To examine if the effect of eCIRP‐mediated impairment of efferocytosis during the early acute phase stroke persists, we analyzed WT and CIRP^−/−^ tMCAO mice on day 3 post‐stroke. As expected, triphenyl tetrazolium chloride (TTC) staining of CIRP^−/−^ tMCAO brains showed reduced infarction at day 3 post‐tMCAO (Figure 2a), with infarct volume significantly decreased by 36.5% compared to WT tMCAO mice (Figure 2b). Additionally, brain levels of IL‐6 were significantly decreased by 74.6% in CIRP^−/−^ mice compared to WT mice on day 3 post‐tMCAO (Figure 2c). CIRP deficiency reduced Bederson scores for neurological deficit on day 3 post‐tMCAO by 50% compared to WT tMCAO mice (Figure 2d). Finally, CIRP^−/−^ mice showed a significantly lower mortality rate of 12.5% compared to 56.3% in WT mice over the course of 3 days following tMCAO (Figure 2e). Collectively, these findings indicate that eCIRP released in IS impairs microglial efferocytic function and worsens stroke outcomes and survival in the acute phase.

### 2.2 ECIRP Induces Pro‐Inflammatory miR‐155 and Suppresses Pro‐Phagocytic Transcription Factor MafB in Ischemic Stroke

**FIGURE 3:**
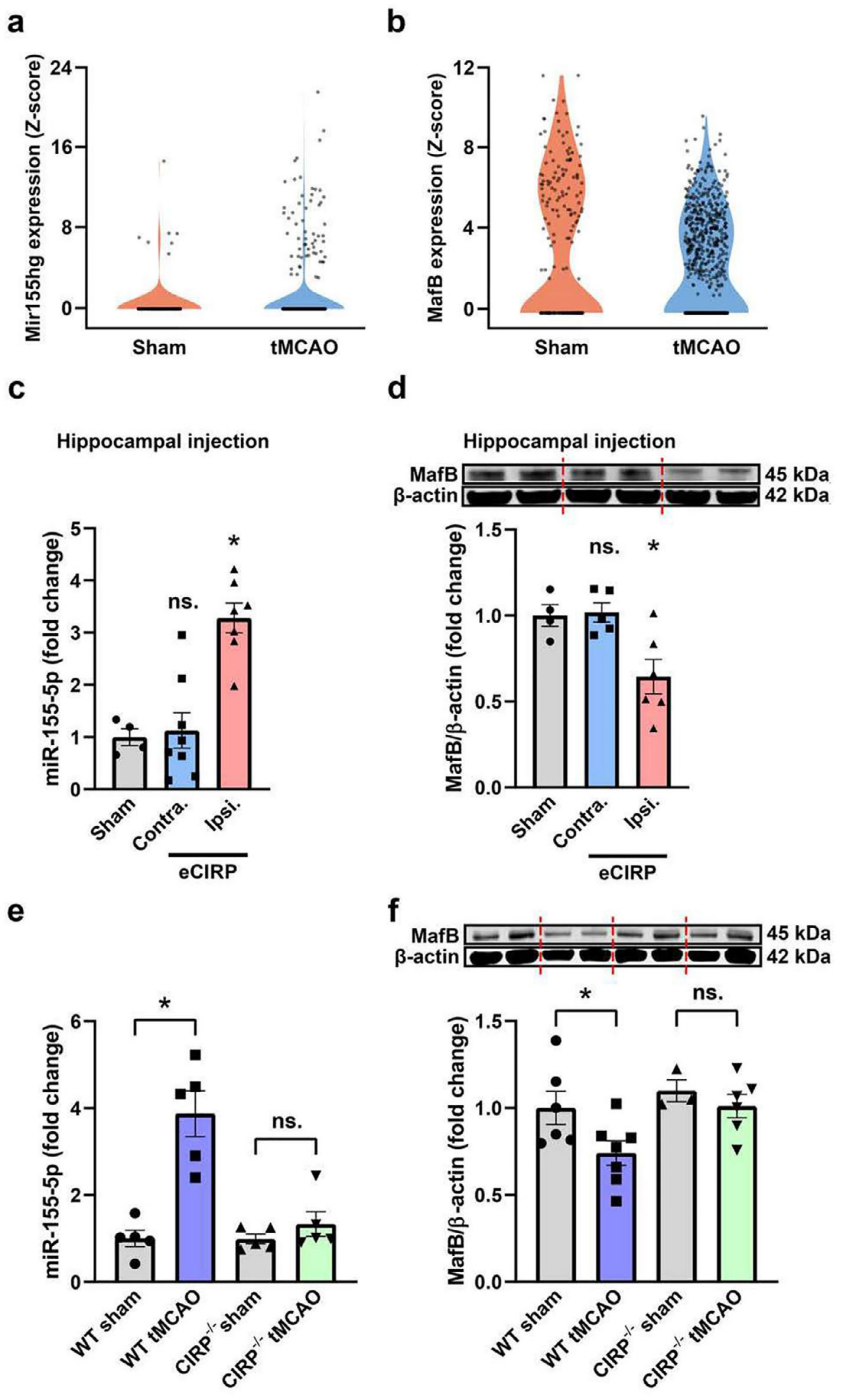
miR‐155 is induced and MafB is suppressed by eCIRP in ischemic stroke. (a,b) miR‐155 host gene (a) *MiR155hg* and (b) *Mafb* expression Z‐scores in microglia cluster from GSE227651. (c) Relative levels of miR‐155 in hippocampus 24 h after stereotactic injection of PBS or 1 µg eCIRP (Contra. & Ipsi. indicate hippocampus laterality relative to eCIRP injection). (*n* = 7–8/group; mean ± SEM; ^*^ *p* = 0.0009 versus Sham & *p* = 0.0002 versus Contra.; ns. *p* = 0.9632 versus Sham; one‐way ANOVA, Tukey's multiple comparisons test). (d) Representative Western blot images and relative MafB protein levels in hippocampal tissues 24 h after stereotactic injection of PBS or 1 µg eCIRP (*n* = 4–6/group; mean ± SEM; ^*^ *p* = 0.0288 versus Sham & *p* = 0.0153 versus Contra.; ns. *p* = 0.989 versus Sham; one‐way ANOVA, Tukey's multiple comparisons test). (e) Relative brain levels of miR‐155 in WT and CIRP^−/−^ mice subjected to sham and tMCAO surgery. (*n* = 5/group; ^*^ *p* = 0.0217 WT sham versus WT tMCAO; ns. *p* = 0.903 CIRP^−/−^ sham versus CIRP^−/−^ tMCAO; two‐way ANOVA, Tukey's multiple comparisons test). (f) Representative Western blot images and relative MafB protein levels normalized to β‐actin in whole brain lysates of WT and CIRP^−/−^ mice subjected to sham and tMCAO surgery. (*n* = 3–7/group; ^*^ *p* = 0.046 WT sham versus WT tMCAO; ns. *p* = 0.7657 CIRP^−/−^ sham versus CIRP^−/−^ tMCAO; Mixed‐effects model (REML) with Sidak's multiple comparisons test).

To investigate the potential mechanism behind eCIRP's microglial efferocytosis‐inhibitory function in IS, we first checked microglial expression of *Mir155hg* and *Mafb* in single‐cell RNA sequencing data set GSE227651 in tMCAO versus sham mouse brains. *Mir155hg*, the long non‐coding RNA host gene for pro‐inflammatory mature miR‐155, was more prevalently and highly expressed in microglia 24 h post‐tMCAO versus sham control (Figure 3a). Accordingly, the expression of *MafB*, a known target of miR‐155‐mediated silencing, decreased in 24 h post‐tMCAO compared to sham (Figure 3b). Furthermore, the microglial *Mir155hg* expression was still higher compared to sham on 3‐day post‐tMCAO brains (Figure S2a). As expected, microglial *MafB* expression in 3‐day post‐tMCAO brains also decreased compared to sham (Figure S2b). Of note the expression changes in microglial *Mir155hg* (Figure S2c) and *MafB* (Figure S2d) were more pronounced at 24 h compared to day 3 post‐tMCAO. To evaluate the specific role of eCIRP in regulating miR‐155 and MafB without other confounding pro‐inflammatory signals present in IS, we stereotactically injected eCIRP into the CA1 hippocampus region of WT mice and harvested hippocampal tissues 24 h post‐injection. Injection of eCIRP increased miR‐155 levels in ipsilateral (injected) hippocampal tissue by 3.3‐fold, but no change was observed in contralateral (non‐injected) or PBS‐injected hippocampus (Figure 3c). MafB protein levels changed inversely; ipsilateral eCIRP‐injected tissues showed significant downregulation of MafB as compared to the contralateral or PBS‐injected tissues (Figure 3d). To test whether eCIRP regulates miR‐155/MafB in IS, we quantified miR‐155 and MafB levels in whole brain lysates collected from WT and CIRP^−/−^ mice subjected to sham or tMCAO surgery. Indeed, miR‐155 increased by 3.8‐fold in WT tMCAO versus WT sham, while CIRP^−/−^ tMCAO mice showed no significant changes compared to CIRP^−/−^ sham mice (Figure 3e). MafB levels showed a clear inverse correlation with miR‐155. MafB protein decreased by 26% in WT tMCAO as compared to WT sham but not in CIRP^−/−^ tMCAO versus CIRP^−/−^ sham (Figure 3f). We further confirmed eCIRP‐induced changes in microglial MafB expression via immunofluorescence co‐staining for Iba‐1 and MafB in tMCAO brain sections, which showed microglial MafB was increased by 2.2‐fold in CIRP^−/−^ tMCAO compared to WT tMCAO (Figure S3). Altogether, these data demonstrate that in IS, microglial miR‐155 is elevated while MafB is decreased, and eCIRP alone is sufficient to mediate this pathway. Furthermore, via eCIRP loss‐of‐function experiments, we show that eCIRP is not only sufficient but necessary to induce miR‐155 and silence MafB.

### 2.3 ECIRP Decreases Microglia Efferocytosis In Vitro via the miR‐155/MafB Axis in a TLR4‐Dependent Manner

**FIGURE 4:**
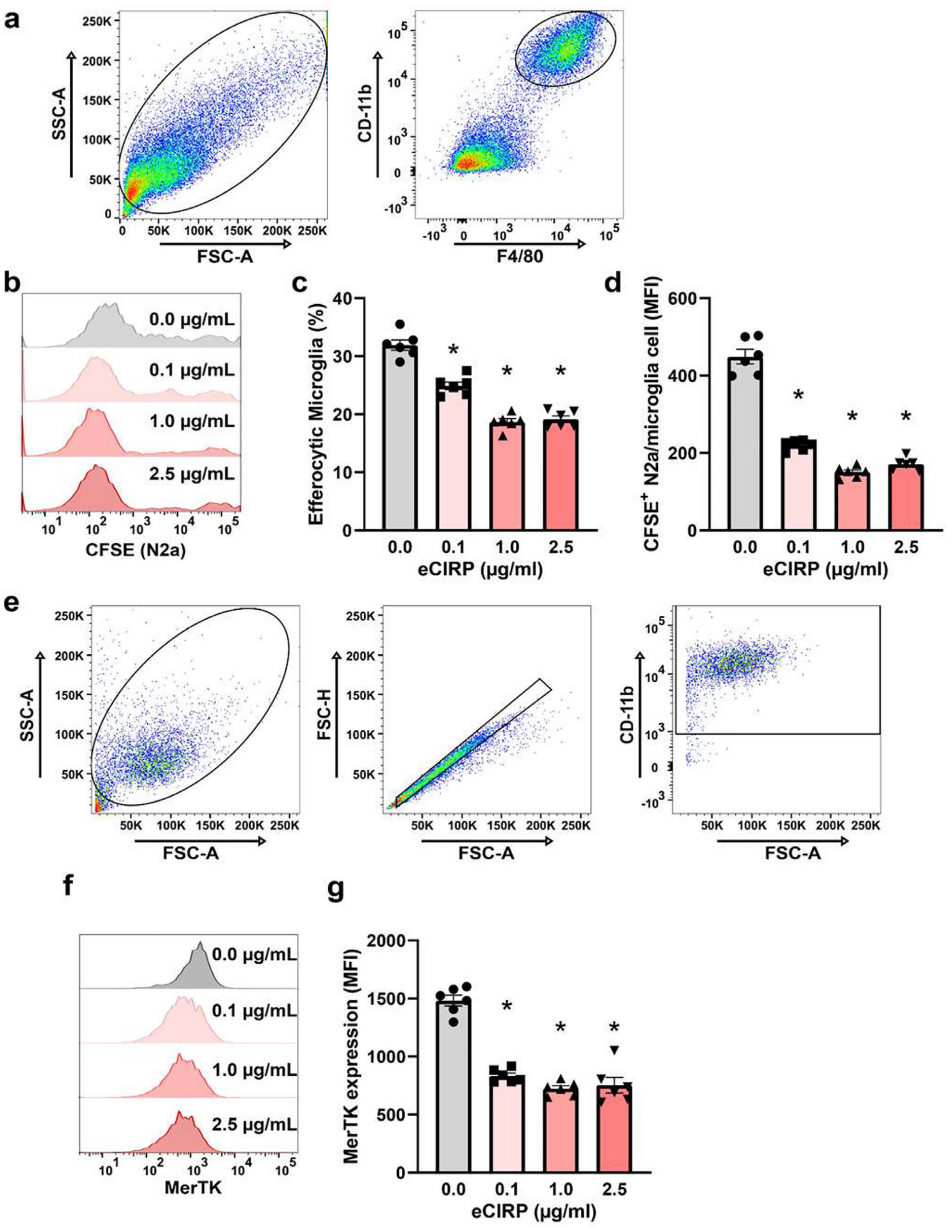
eCIRP causes microglial efferocytic dysfunction in vitro. (a–d) Primary microglia were stimulated with indicated eCIRP doses for 20 h and incubated with CFSE labeled dead N2a neurons at 1:3 ratio for 1 h, and efferocytosis was measured via flow cytometry. (a) Gating strategy for microglia in mixed microglia/N2a samples. (b) Representative histograms of N2a CFSE signal in CD11b^+^F4/80^+^ microglia populations at indicated doses of eCIRP. (c) Percentage of efferocytic CFSE^+^/CD‐11b^+^/F4/80^+^ microglia and (d) Median fluorescence intensity (MFI) of CFSE in microglia at increasing eCIRP doses. (*n* = 6/group; mean ± SEM ^*^ *p* < 0.0001 versus 0 µg/mL; one‐way ANOVA, Tukey's multiple comparisons test). (e–g) Primary microglia were stimulated with indicated eCIRP doses for 20 h, and MerTK staining was assessed via flow cytometry. (e) Gating strategy for pure microglia samples. (f) Representative histograms of MerTK signal in CD‐11b^+^ gated microglia at indicated doses of eCIRP. (g) MerTK MFI of microglia at increasing eCIRP doses. (*n* = 6/group; mean ± SEM; ^*^ *p* < 0.0001 versus 0 µg/mL; one‐way ANOVA, Tukey's multiple comparisons test).

To gain more insight into eCIRP‐mediated efferocytic dysfunction, we established in vitro cultures of primary microglia isolated from neonatal mice. First, we conducted in vitro efferocytosis assays using primary microglia stimulated for 20 h with increasing concentrations of eCIRP (0, 0.1, 1, and 2.5 µg/mL), followed by co‐incubation with CFSE stained, UV‐killed, murine Neuro‐2a (N2a) neuronal cells and analyzed using flow cytometry. eCIRP significantly reduced the percentage of efferocytic microglia in a dose‐dependent manner, with 1 µg/mL eCIRP decreasing the efferocytic microglia by 13.3%, a relative decrease of 41.5% (Figure 4a–c). Importantly, eCIRP also significantly reduced the amount of N2a cells engulfed per microglia in a dose‐dependent manner, with 1 µg/mL eCIRP decreasing the MFI of CFSE‐stained N2a cells in microglia by 66.5% (Figure 4d). These findings were confirmed with an imaging‐based efferocytosis assay, utilizing UV‐killed biotinylated N2a cells co‐incubated with eCIRP‐stimulated or control microglia and FITC‐streptavidin staining to distinguish internalized versus externalized efferocytosis cargo. Indeed, eCIRP‐treated microglia showed a qualitative decrease in N2a efferocytosis (Figure S4). We also verified eCIRP‐induced MerTK downregulation in vitro using flow cytometry (Figure 4e,f). eCIRP downregulated the cell surface MerTK expression in primary microglia in a dose‐dependent manner, with 1 µg/mL eCIRP downregulating surface MerTK by 51.2% (Figure 4g). Furthermore, *Mertk* gene expression in eCIRP stimulated microglia was also decreased by 51.8% compared to untreated controls (Figure S5a).

**FIGURE 5:**
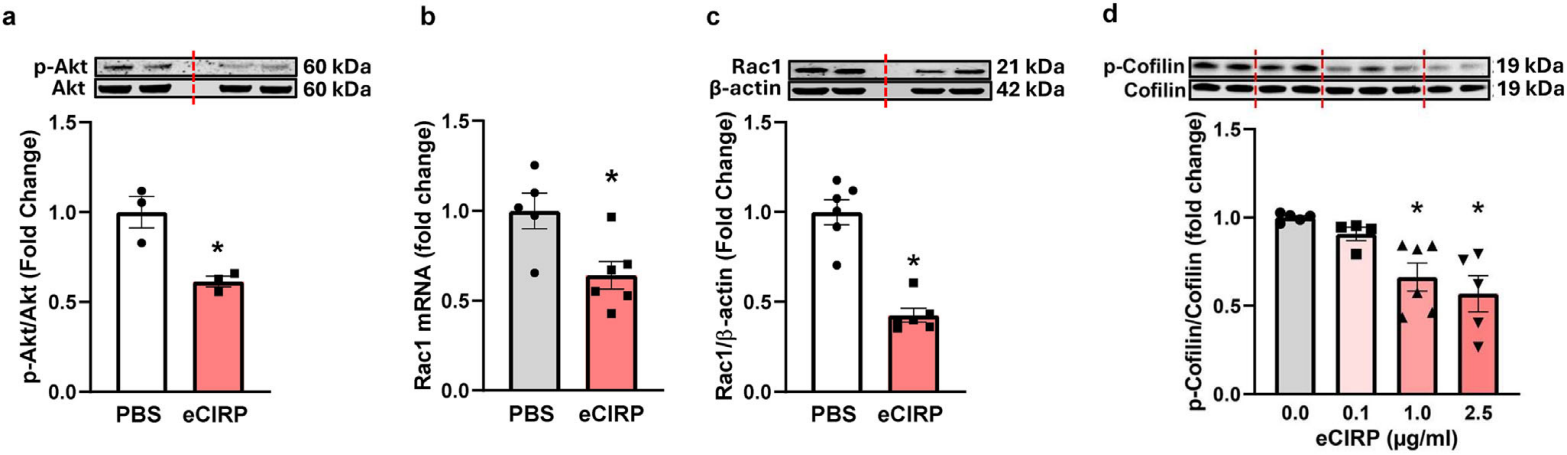
eCIRP regulates downstream intracellular efferocytosis mediators in primary microglia in vitro. Primary microglia were stimulated with PBS or eCIRP (1 µg/mL) for 20 h. (a) Representative Western Blot images and quantification of phosphorylated Akt (p‐Akt) normalized to total Akt protein levels in microglia (*n* = 3/group; ^*^ *p* = 0.0143; Unpaired *t*‐test). (b) RT‐qPCR quantification of microglial Rac1 mRNA expression (*n* = 5–6/group; ^*^ *p* = 0.0173; Unpaired *t*‐test). (c) Microglial Rac1 protein expression shown as representative Western Blot images and quantification normalized to β‐actin (*n* = 6/group; ^*^ *p* < 0.0001; Unpaired *t*‐test). (d) Representative Western Blot images and quantification of p‐Cofilin normalized to total Cofilin protein levels in microglia treated with indicated doses of eCIRP for 20 h. (*n* = 4–6/group; mean ± SEM; ^*^ *p* = 0.0156 1 µg/mL versus 0 µg/mL and ^*^ *p* = 0.0032 2.5 µg/mL versus 0 µg/mL; one‐way ANOVA, Tukey's multiple comparisons test).

Since eCIRP is known to bind to TLR4, we examined whether TLR4 was required for MerTK downregulation in microglia. Therefore, we isolated primary microglia from WT and TLR4^−/−^ neonatal mice and quantified MerTK mRNA levels. In contrast to WT microglia, TLR4^−/−^ microglia did not significantly downregulate MerTK in response to eCIRP stimulation (Figure S5b). We then sought to uncover whether eCIRP's downregulation of MerTK has any effect on activation of intracellular efferocytosis machinery, specifically cytoskeletal remodeling regulators. For this, we assessed the activation status of downstream PI3K/Akt signaling, Rac1 expression, and cofilin phosphorylation in eCIRP‐treated microglia. eCIRP markedly decreased activation of PI3/Akt signal downstream of MerTK shown as 38.53% downregulation in microglial levels of phosphorylated Akt compared to control (Figure 5a). eCIRP stimulation significantly diminished microglial Rac1 mRNA levels by 35.8% (Figure 5b), and microglial Rac1 protein levels by 57.38% (Figure 5c). Microglial p‐Cofilin decreased with eCIRP treatment in a dose‐dependent manner, with 1 µg/mL eCIRP downregulating cofilin phosphorylation by 33.8% (Figure 5d). These data suggest that eCIRP causes defects in actin dynamics necessary for efficient efferocytosis.

**FIGURE 6:**
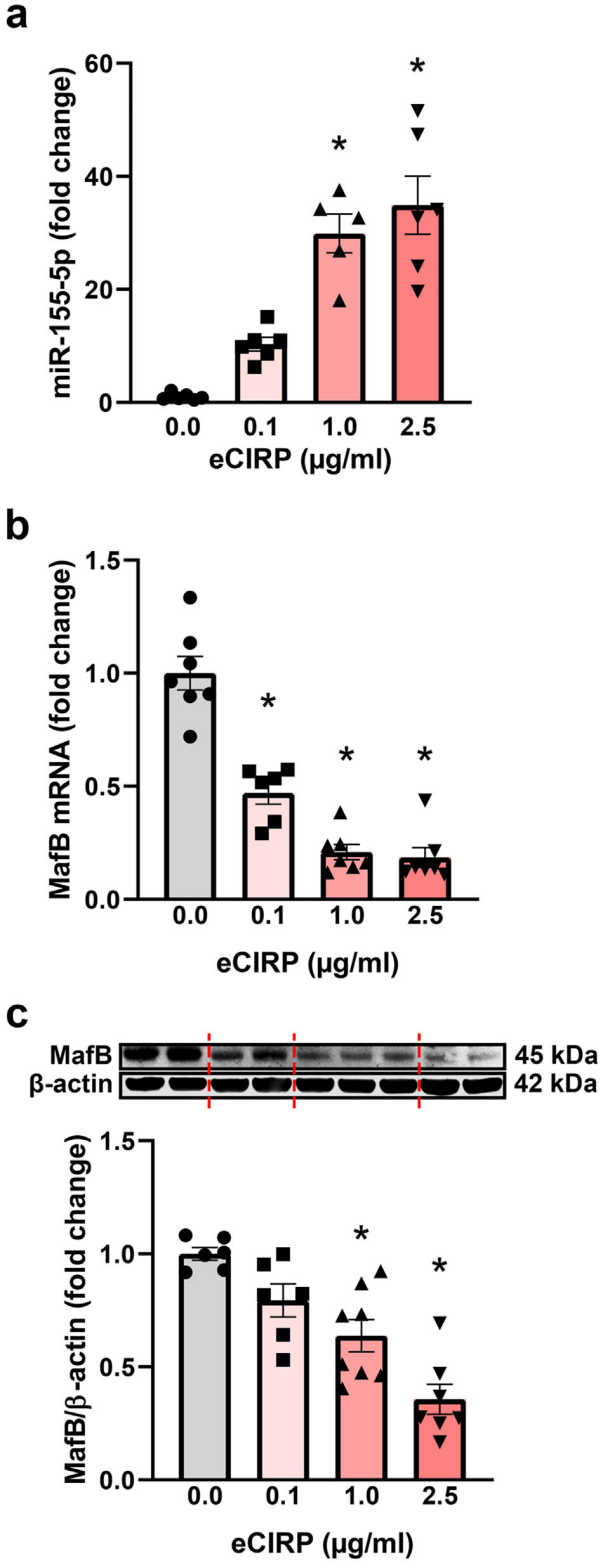
eCIRP upregulates miR‐155 and downregulates MafB in primary microglia in vitro. Primary microglia were treated with indicated eCIRP doses for 20 h prior to RNA or protein isolation. (a) RT‐qPCR quantification of relative miR‐155 expression. (*n* = 5–6/group; mean ± SEM; ^*^ *p* < 0.0001 versus 0 µg/mL; one‐way ANOVA, Tukey's multiple comparisons test). (b) RT‐qPCR quantification of relative *Mafb* gene expression. (*n* = 6–7/group; mean ± SEM ^*^ *p* < 0.0001 versus 0 µg/mL; one‐way ANOVA, Tukey's multiple comparisons test). (c) Representative Western Blot image and quantification of MafB protein expression normalized to β‐actin. (*n* = 6–8/group; mean ± SEM; ^*^ *p* = 0.0031 1 µg/mL versus 0 µg/mL and ^*^ *p* < 0.0001 2.5 µg/mL versus 0 µg/mL; one‐way ANOVA, Tukey's multiple comparisons test).

**FIGURE 7:**
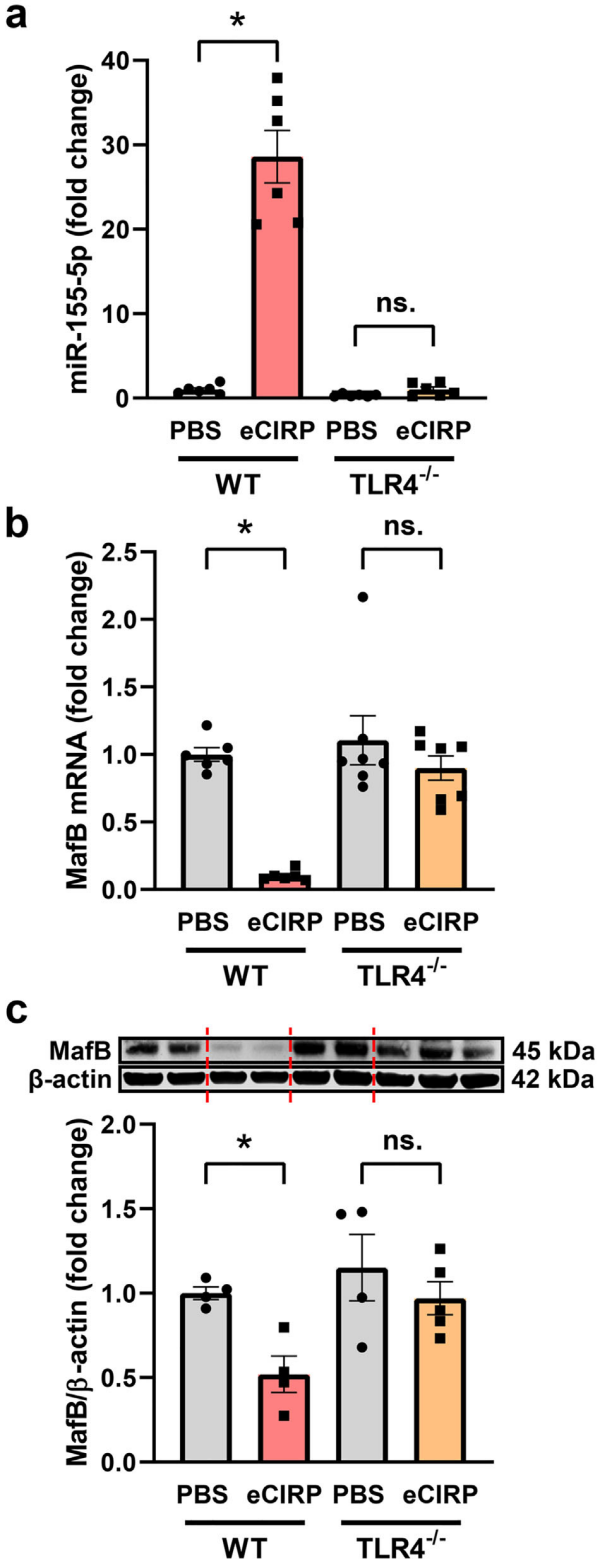
TLR4 is necessary for eCIRP‐mediated regulation of miR‐155 and MafB. Primary microglia were isolated from WT and TLR4^−/−^ neonatal mice and treated with PBS or 1 µg/mL eCIRP for 20 h. (a) RT‐qPCR quantification of relative miR‐155 expression in WT and TLR4^−/−^ microglia. (*n* = 6/group; mean ± SEM; ^*^ *p* = 0.0003 WT eCIRP versus WT PBS, ns. *p* = 0.9892 TLR4^−/−^ eCIRP versus TLR4^−/−^ PBS; two‐way ANOVA, Sidak's multiple comparisons test). (b) RT‐qPCR quantification of relative *Mafb* gene expression in WT and TLR4^−/−^ microglia. (*n* = 6–7/group; mean ± SEM; ^*^ *p* = 0.019 WT eCIRP versus WT PBS, ns. *p* = 0.5838 TLR4^−/−^ eCIRP versus TLR4^−/−^ PBS; Mixed‐effects model (REML) with Sidak's multiple comparisons test). (c) Representative Western Blot images and quantification of MafB protein expression in WT and TLR4^−/−^ microglia normalized to β‐actin. (*n* = 4–5/group; mean ± SEM; ^*^ *p* = 0.0399 WT eCIRP versus WT PBS control, ns. *p* = 0.3991 TLR4^−/−^ eCIRP versus TLR4^−/−^ PBS control; Mixed‐effects model (REML) with Sidak's multiple comparisons test).

Next, using primary microglia we verified the mechanism by which eCIRP transcriptionally reprograms the microglia to induce efferocytic dysfunction. eCIRP increased miR‐155 dose‐dependently, with 29.9‐fold induction at 1 µg/mL and 34.9‐fold induction at 2.5 µg/mL relative to baseline (Figure 6a). Conversely, eCIRP significantly downregulated MafB mRNA levels in a dose‐dependent manner, with a 77.9% decrease from the baseline expression at 1 µg/mL (Figure 6b). MafB protein levels had a similar pattern and decreased by 36.2% and 63.3% at 1 and 2.5 µg/mL eCIRP respectively (Figure 6c). Of note, eCIRP also diminished the mRNA expression of MafB transcriptional target C1q by 94.6% at 1 µg/mL, verifying that MafB activity was also significantly suppressed (Figure S6). Additionally, BV‐2 cells, an immortalized murine microglial cell line, transfected with a miR‐155 mimic expressed less MafB protein relative to control mimic with levels comparable to eCIRP stimulated BV‐2 cells, confirming miR‐155's role in translational repression of MafB (Figure S7). Since induction of miR‐155 in brain has been shown to be TLR4‐dependent [39], we examined whether TLR4 was required for eCIRP to initiate miR‐155 expression and resultant MafB suppression in microglia. In contrast to WT primary microglia, TLR4^−/−^ microglia did not significantly upregulate miR‐155 when stimulated with eCIRP (Figure 7a). Accordingly, unlike WT microglia TLR4^−/−^ microglia also did not downregulate MafB mRNA (Figure 7b) or MafB protein levels (Figure 7c). These data collectively show that eCIRP induces microglial efferocytosis dysfunction via TLR4/miR‐155/MafB axis.

### 2.4 Targeted Inhibition of eCIRP/TLR4 Binding with Small Peptide C23 Rescues Efferocytic Dysfunction in Ischemic Stroke

**FIGURE 8:**
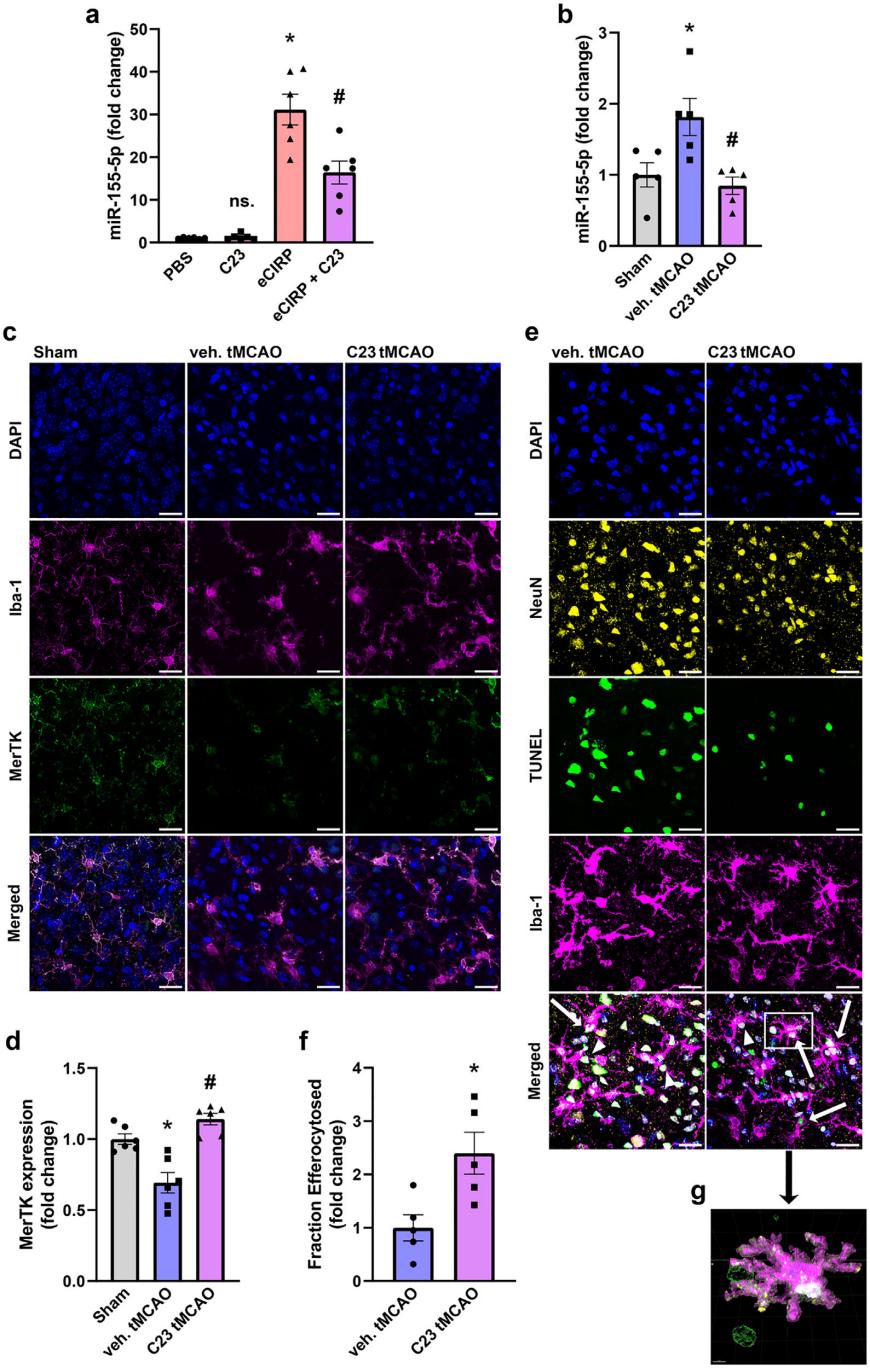
C23, a small peptide inhibitor of eCIRP/TLR4 binding, attenuates miR‐155 induction in vitro and in vivo and rescues MerTK expression and microglial efferocytosis in ischemic stroke. (a) Primary microglia were treated with C23 (1 µg/mL), eCIRP (1 µg/mL), or a mixture of eCIRP and C23 for 20 h, and miR‐155 expression was quantified via RT‐qPCR. (*n* = 6/group; mean ± SEM; ^*^ *p* < 0.0001 versus PBS, ^#^ *p* = 0.0008 eCIRP + C23 versus eCIRP; one‐way ANOVA, Tukey's multiple comparisons test). (b–g) WT mice were subjected to sham or tMCAO surgery and received retroorbital injection of C23 or vehicle solution (veh., PBS). (b) RT‐qPCR quantification of relative miR‐155 levels in whole brain lysates of Sham, veh. tMCAO, and C23 tMCAO mice. (*n* = 5/group; mean ± SEM; ^*^ *p* = 0.0298 veh. tMCaO versus Sham; ^#^ *p* = 0.0109 C23 tMCAO versus veh. tMCAO; one‐way ANOVA, Tukey's multiple comparisons test). (c) Representative confocal images of peri‐infarct brain tissues in Sham, veh. tMCAO and C23 tMCAO mice, stained for DAPI (blue), Iba‐1 (magenta), and MerTK (green). Magnification 630x, scale bar, 20 µM. (d) Quantification of MerTK expression in microglia. (*n* = 6/group; mean ± SEM; ^*^ *p* = 0.0022 veh. tMCAO versus Sham; ^#^ *p* < 0.0001 C23 tMCAO versus veh. tMCAO; one‐way ANOVA, Tukey's multiple comparisons test). (e) Representative confocal images of peri‐infarct brain tissues in veh. tMCAO and C23 tMCAO mice, with staining for nuclei (DAPI, blue), neurons (NeuN, yellow), dead/dying cells (TUNEL, green), and microglia (Iba‐1, magenta); arrows indicate efferocytosis events with complete internalization of TUNEL^+^/NeuN^+^ cargo, arrowheads indicate incomplete efferocytosis events. Magnification 630x, scale bar, 20 µM. (f) Ratio of TUNEL^+^ neurons completely engulfed by microglia to total TUNEL^+^ neurons expressed as fold‐change with veh. tMCAO set as 1. (*n* = 5/group; mean ± SEM; ^*^ *p* = 0.0168; Unpaired *t*‐test). (g) Zoomed in high power reconstruction of 3D segmented surfaces in Imaris showing efferocytosis event indicated in white box in **e**, demonstrating TUNEL^+^/NeuN^+^ cargo engulfed by Iba‐1^+^ microglia.

After characterizing the mechanism of eCIRP‐mediated microglial efferocytic dysfunction, we tested a therapeutic approach to antagonize eCIRP/TLR4 interaction. First, we treated microglia with C23, eCIRP, or a mixture of eCIRP and C23 (eCIRP + C23) and compared miR‐155 expression across these groups. C23 alone did not affect basal miR‐155 levels of primary microglia, and as expected, eCIRP induced miR‐155 expression by 31.2‐fold (Figure 8a). Indeed, co‐treatment with C23 and eCIRP attenuated eCIRP‐induced miR‐155 expression by 47.3% (Figure 8a). To test C23's effect in vivo, mice were subjected to tMCAO and retro‐orbitally injected at the time of reperfusion with C23 (10 µg/g b.w.) or PBS vehicle solution (veh.). Brain levels of miR‐155 were increased by 1.8‐fold in veh. tMCAO mice compared to sham mice, while C23 treatment significantly attenuated the miR‐155 levels in tMCAO mice, bringing them down to sham levels, (Figure 8b). Microglial MerTK expression measured by immunofluorescence co‐staining with Iba‐1 and MerTK (Figure 8c), demonstrated a 30.7% decrease in MerTK expression in veh. tMCAO brain tissue with respect to sham control (Figure 8d). However, C23 treatment completely abrogated this reduction in MerTK expression (Figure 8c,d). Subsequently, we assessed efferocytosis in peri‐infarct regions of tMCAO brains via staining for Iba‐1, NeuN, and DAPI, followed by TUNEL labeling (Figure 8e), and 3D‐segmentation analysis in Imaris (Figure 8g), which showed a 2.4‐fold increase in microglial efferocytosis in the C23 treated group compared to vehicle treated tMCAO (Figure 8f). Thus, C23 attenuates eCIRP‐mediated miR‐155 induction in vitro and in vivo, and improves microglial expression of MerTK and rescues microglial efferocytic dysfunction in IS.

### 2.5 C23 Treatment Improves Ischemic Stroke Outcome

**FIGURE 9:**
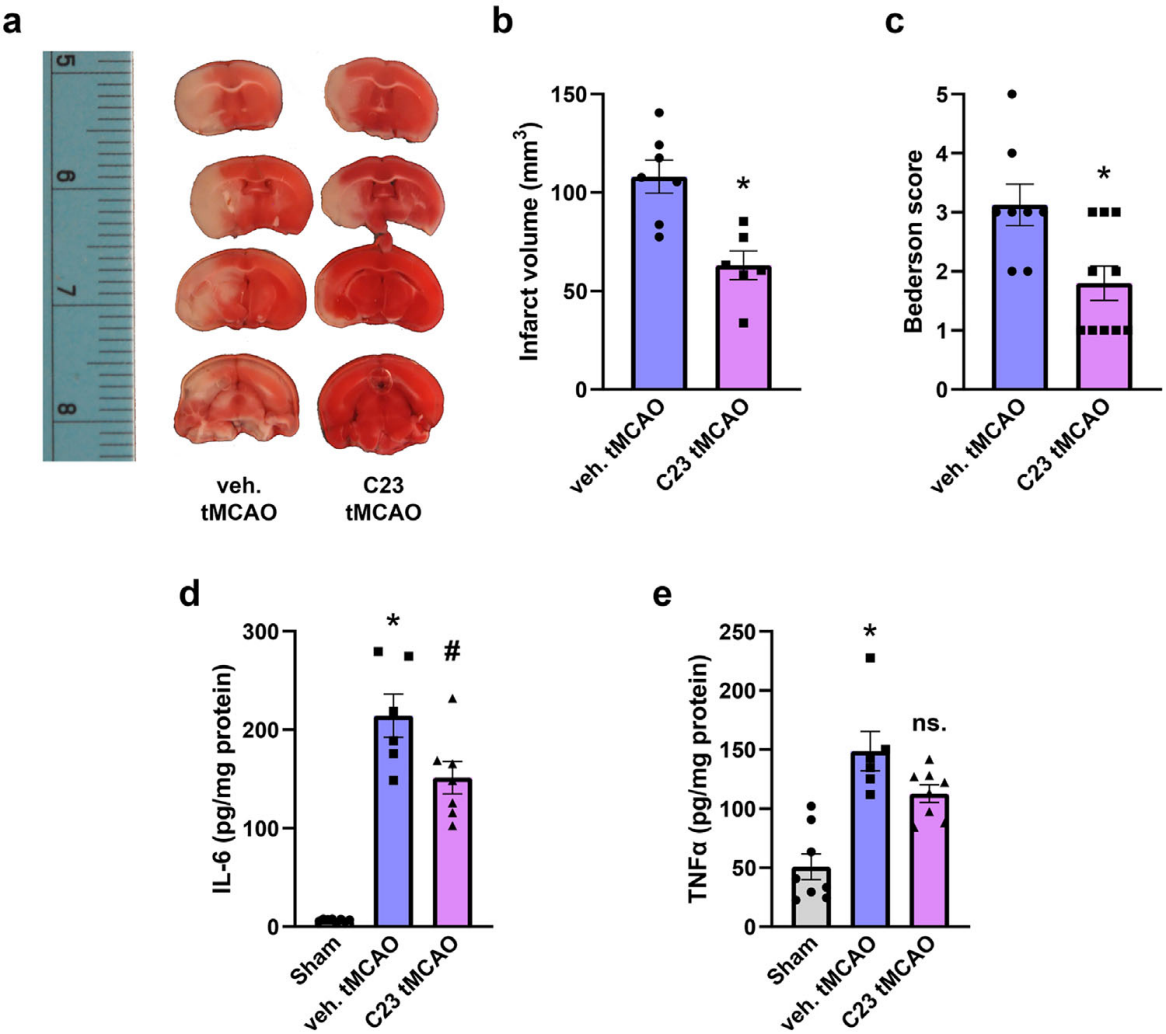
C23 treatment improves outcomes in murine ischemic stroke. WT mice were subjected to Sham or tMCAO surgery and received retroorbital injection of C23 or vehicle solution (veh., PBS). (a) Representative images of 2 mm thick brain sections stained with TTC for infarction detection and quantification. Infarcted brain tissue is stained pale white, while healthy brain tissue is stained in red. (b) Infarct volume in veh. or C23 treated mice 24 h after tMCAO quantified with NIH ImageJ. (*n* = 6–7/group; mean ± SEM; ^*^ *p* = 0.0021; Unpaired *t*‐test). (**c)** Bederson score 24 h after tMCAO in veh. versus C23 treated mice (*n* = 8–10/group; mean ± SEM; ^*^ *p* = 0.0097; Unpaired *t*‐test). (d,e) 24 h post‐reperfusion, whole brains were collected and protein isolated. (d) IL‐6 tissue ELISA expressed as pg IL‐6/mg total protein. (*n* = 6–8/group; ^*^ *p* < 0.0001 versus Sham; ^#^ *p* = 0.02 versus veh. tMCAO; one‐way ANOVA, Tukey's multiple comparisons test). (**e)** TNFα tissue ELISA expressed as pg TNFα/mg total protein. (*n* = 6–8/group; ^*^ *p* < 0.0001 versus Sham; ns. *p* = 0.1056 versus veh. tMCAO; one‐way ANOVA, Tukey's multiple comparisons test).

We then sought to evaluate the efficacy of C23 in improving outcome measurements as a neuroprotective and immunomodulatory agent in IS. We evaluated acute outcomes 24 h post‐tMCAO after a one‐time dose of C23 injected retro‐orbitally. TTC staining of fresh brain sections was conducted exhibiting a decrease in infarction in tMCAO mice that received C23 compared to mice treated with vehicle (Figure 9a). Quantification of the infarct volume showed a significant reduction by 41.7% in C23 treated tMCAO mice compared to veh. treated tMCAO mice (Figure 9b). C23 was also successful in reducing neurological deficit with C23 treated mice exhibiting forelimb flexion and decreased resistance to lateral push while vehicle‐treated mice exhibited unidirectional circling, shown as 42.3% decrease in Bederson score in the C23 tMCAO mice (Figure 9c). In addition, C23 significantly attenuated brain levels of IL‐6 by 29.4% versus veh. treatment (Figure 9d), and TNFα levels tended to decrease compared to veh. tMCAO (Figure 9e). The tMCAO procedure used in this study is too severe for long‐term survival study. Indeed, in a very small cohort tested (n = 4/group) tMCAO mice administered with vehicle resulted in 100% mortality over the course of 7 days. Notably, C23 administration lowered the mortality rate to 25% in tMCAO mice (Figure S8). Thus, our findings show that C23 is a potential neuroprotective therapy candidate that limits infarction, inflammation, and behavioral deficit in ischemic stroke, which warrants further evaluation.

## 3 Discussion

The emerging role of eCIRP, in post‐stroke neuroinflammation is intriguing and needs to be completely elucidated. Efferocytosis is a relatively new area of study that is garnering increasing attention in the field of IS pathology and drug development [22, 40, 41]. Microglia and infiltrating peripheral macrophages have been shown to conduct efferocytosis in IS [40, 42, 43]. eCIRP activates microglia and shifts microglia to a “classical” pro‐inflammatory phenotype via NF‐κB [12, 15]. However, it remains to be determined whether eCIRP regulates microglial efferocytosis in IS, if so, what specific molecular mechanisms are involved. Discovery of molecular targets of eCIRP associated with microglial efferocytosis could lead to novel targeted therapies capable of enhancing efferocytosis in IS.

**FIGURE 10:**
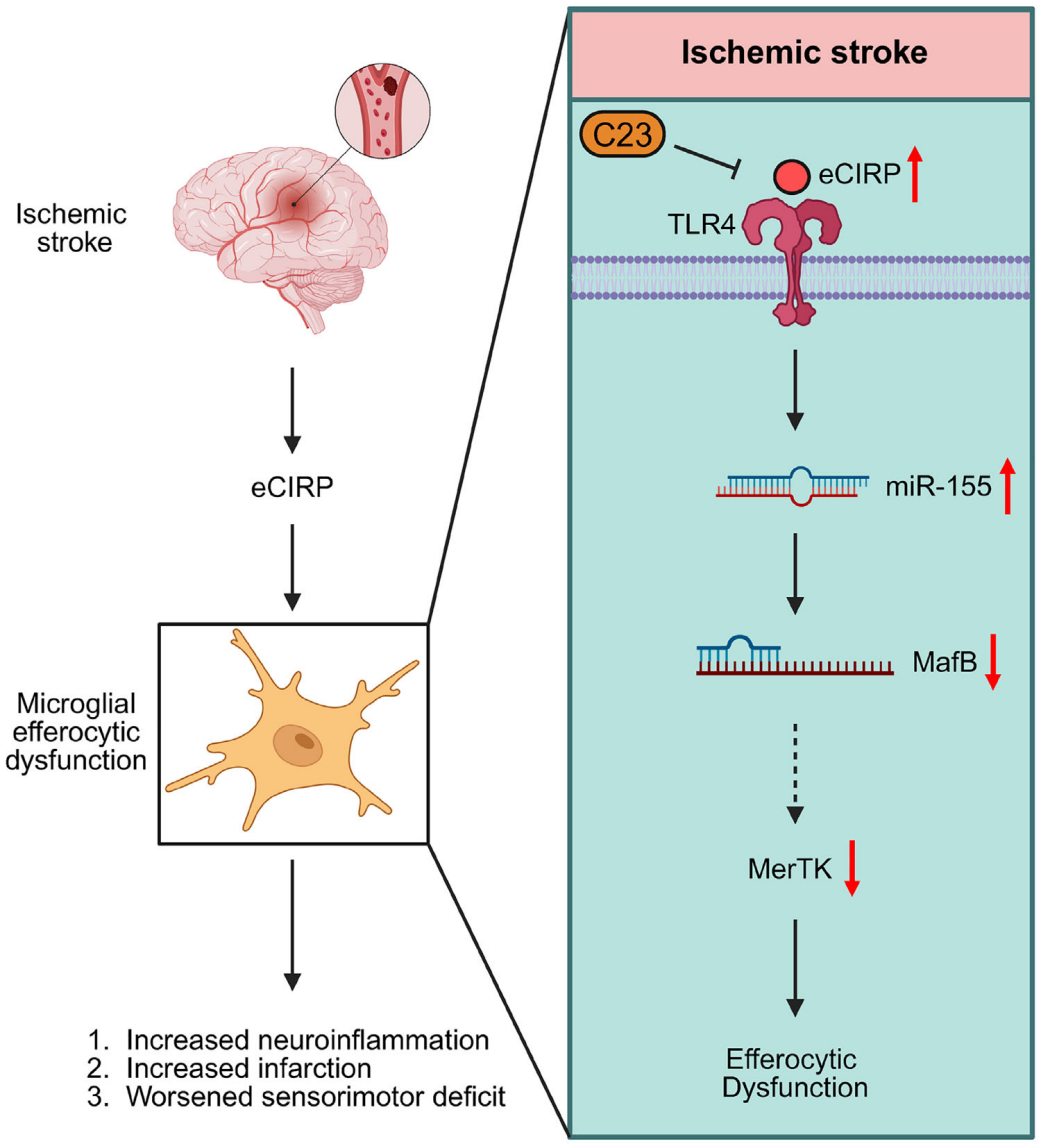
Schematic diagram summary. eCIRP is released in stroke brain and binds to TLR4 expressed on microglia and induces miR‐155 and suppresses MafB, causing decreased MerTK expression and impaired efferocytosis. In addition, inhibition of eCIRP‐TLR4 interaction with small peptide C23 attenuates eCIRP‐induced microglial miR‐155 expression and downstream microglial efferocytic dysfunction.

Herein, we report that eCIRP via TLR4 binding regulates the miR‐155/MafB pathway, induces MerTK downregulation and microglial efferocytic dysfunction in IS, and targeting this interaction with small inhibitory peptide C23 improves acute IS outcomes (Figure 10). Microglia‐specific phagocytic pathway activities in the spatial context of an ischemic brain injury tend to be masked by cellular heterogeneity in generalized bulk analyses of whole brain tissue. Thus, we utilized tissue immunofluorescence microscopy in vivo, which directly assessed and quantified the microglia‐specific efferocytosis function and MerTK expression in the peri‐infarct brain region. First utilizing the tMCAO model of IS, we showed that eCIRP is released into CSF and microglial MerTK expression is decreased in vivo 24 h post‐stroke. Next, using tMCAO in CIRP‐deficient mice, we showed that eCIRP diminishes MerTK expression, impairs microglial efferocytosis function 24 h post‐stroke, and worsens the acute IS outcomes 3 days post‐stroke. We then illustrated that microglial miR‐155 expression was increased, while microglial MafB expression was markedly reduced 24 h post‐tMCAO. This inverse association in miR‐155 and MafB expression was then recapitulated at the site of hippocampal eCIRP injection. In contrast, CIRP^−/−^ tMCAO mice had attenuated miR‐155, but preserved MafB expression in the brain compared to WT counterparts. We further investigated this mechanism in vitro, showing that eCIRP downregulated microglial efferocytosis receptor MerTK and its downstream signaling mediators involved in cytoskeletal rearrangements. Moreover, eCIRP induced efferocytosis dysfunction via the miR‐155/MafB axis in a TLR4‐dependent manner. Furthermore, we demonstrated that C23, a small peptide blocking eCIRP‐TLR4 interaction, rescued microglial efferocytic dysfunction in tMCAO by attenuating miR‐155 and increasing MerTK. C23 reduced levels of inflammatory cytokines, infarct volume and neurological impairment in tMCAO indicating improved acute stroke outcome.

There is a marked increase in CSF eCIRP 24 h after tMCAO in our study. Of note, a recent study showed in vivo that immunostaining for CIRP in the ischemic penumbra significantly increased primarily in neurons and in some microglia at 24 h after tMCAO, while it did not change much in astrocytes or endothelial cells [14]. In the same study, primary neurons subjected to oxygen glucose deprivation/re‐oxygenation in vitro for 6 h also induced release of eCIRP into culture medium [14]. Moreover, we have previously demonstrated that BV‐2 microglial cells subjected to hypoxia in vitro for 20–30 h significantly upregulated the release of eCIRP into culture medium [12]. Therefore, in line with the published literature it is most likely that in our study neurons are the primary source of eCIRP released into tMCAO CSF along with some contribution from activated microglia. A recent study in tMCAO mice showed that CIRP levels in the ischemic penumbra in the brain during ischemic stroke start increasing at 6 h, significantly increased at 24 h, peaking at day 3, then subsiding a little but still persisting at day 7 [14]. Interestingly, the eCIRP levels in the serum of acute ischemic stroke patients also showed similar dynamics and higher serum eCIRP levels correlated with increased stroke‐associated disability on admission that persisted even after 3‐month follow up [17]. This suggests persistence of consequences of the acute increase in eCIRP levels into the later subacute phases. Interestingly, the miR‐155/MafB axis remained impaired on day 3 post‐stroke, although not as pronounced as on 24 h after stroke. Since efferocytosis and microglial phenotypes are also highly dynamic and interconnected processes, it is imperative for future studies to take into consideration whether the eCIRP/TLR4‐mediated impairment of miR‐155/MafB axis during the acute stroke continues in the later phases or resolves over time. If the eCIRP release dampens after early‐phase, the miR‐155/MafB axis would revert to transitioning microglia into the pro‐efferocytic, pro‐resolving phenotype required for the repair in subacute and chronic phases. Thus, targeting eCIRP during the acute phase would be beneficial by limiting the harmful microglial efferocytic dysfunction that prolongs inflammation worsening the brain injury and functional recovery after stroke.

The role of miR‐155 is widely studied and this proinflammatory miRNA is known to be induced in ischemic brain injury [32, 33]. Mechanistically, dual luciferase reporter assay showed that miR‐155 directly targeted 3’UTR of MafB to silence MafB translation and suppressed MafB expression [32]. Our work demonstrates that eCIRP is a potent inducer of miR‐155 in vitro and in vivo, and furthermore suggests that eCIRP may be the main inducer in tMCAO. eCIRP precipitously and dose‐dependently decreased microglial MafB at the mRNA and protein levels in vitro. MafB was also downregulated by eCIRP in vivo. We show that eCIRP's effects on miR‐155 and MafB are dependent on microglial expression of TLR4 corroborating previous work on miR‐155, which demonstrated that TLR4 is necessary for miR‐155 induction in the brain [39, 44]. We also showed that similar to eCIRP stimulation, miR‐155 overexpression in BV2 cells was sufficient to suppress MafB expression. To further strengthen and validate the eCIRP regulation of prior established miR‐155/MafB axis, more detailed mechanistic studies are warranted. The literature clearly supports that loss of function or early inhibition of miR‐155 improves IS outcomes [32, 33, 44, 45, 46]. In contrast, distal MCAO mice treated with a miR‐155 inhibitor 48 h post‐stroke exhibited decreased expression of CD68, a phagocytic marker, in peri‐vascular microglia and macrophages [47]. Together, these data suggest that miR‐155 is an important inducer of post‐stroke inflammation that amplifies tissue‐degenerative early neuroinflammation, as we demonstrate here, but may also play a role in later‐phase tissue restorative neuroinflammation. Accordingly, miR‐155's target, MafB is known to be anti‐inflammatory and pro‐phagocytic. Interpretation of the differential expression of these phagocytosis‐related factors using GO/KEGG pathway or gene set enrichment analysis studies in future will help gain additional mechanistic insights in the eCIRP regulation of microglial phagocytosis and further validate its functional relevance. MafB is well characterized to promote phagocytosis and efferocytosis in peripheral macrophage cell lines [48, 49]. Though our experiments focused on microglial efferocytosis, given the convergent nature of intracellular signaling and efferocytic function in microglia and macrophages, it is very likely that eCIRP may also regulate the TLR4/miR‐155/MafB pathway in the macrophages infiltrating the brain in later time points following an ischemic stroke. Accordingly, we expect C23 to also improve the efferocytic function and dampen the inflammatory function of the infiltrating macrophages in addition to its effect on improving earlier defects in microglial efferocytosis. Due to the relevance of infiltrating macrophages in the ischemic stroke injury and recovery, it would be interesting to further explore the effect of eCIRP and C23 on infiltrating macrophages independently taking into consideration the timing and complexity of their dynamic interaction. MafB has also been shown to augment DAMP scavenging by infiltrating peripheral macrophages in IS [29]. Furthermore, activation of MafB with a synthetic retinoid reduced infarct volume and neurological deficit from 4–28 d post injury supporting its tissue‐restorative role [29]. Broadly, MafB is emerging as a critical transcription factor for adopting the tissue‐reparative phenotype in microglia and macrophages [30, 31]. Hence, eCIRP‐mediated MafB silencing is likely to impair the tissue‐restorative functions of microglia and macrophages beyond its impact on efferocytosis.

eCIRP‐induced functional impairment in microglial efferocytosis was validated in vitro utilizing flow cytometry, microscopic imaging, and assessing key signaling pathway mediators. We showed that eCIRP significantly decreased MerTK, which is crucial for microglial efferocytosis. MerTK directly regulates microglial efferocytosis by driving phagocytic cup closure for engulfment of apoptotic cells [50]. This suggests MerTK to be the primary driver of eCIRP‐induced efferocytic dysfunction. We have previously shown that eCIRP impairs bacterial phagocytosis in macrophages and dissected the downstream phagocytosis pathways involving Rac1 activation and actin remodeling in detail [36]. In the context of eCIRP regulation of MerTK‐mediated microglial efferocytosis, we also identified PI3K/Akt activation, Rac1 and cofilin regulation of actin to be downstream mediators. These data suggest generalized convergence of eCIRP‐mediated downstream signaling in the contexts of macrophage phagocytosis and microglial efferocytosis.

Of note, we also found that eCIRP dramatically suppressed C1q, a transcriptional target of MafB [51]. This precipitous decrease in microglial C1q expression is most likely due to miR‐155‐mediated MafB loss, as MafB has been shown to be the chief regulator of C1q transcription in macrophages [51]. It is, however, possible that other mechanisms downstream of eCIRP could mediate this effect. Canonically, C1q is the initiator of the classical complement pathway functioning to opsonize or lyse exogenous pathogens [52]. However, recently, C1q has been shown to augment efferocytosis, promote MerTK expression, and inhibit pro‐inflammatory cytokine secretion in macrophages [53, 54, 55, 56]. This mechanism is not yet well understood, but an AMPKα‐mediated mechanism has been proposed [55]. Since classically C1q acts as an opsonin and enhances recognition by tagging target cells, the suppression of C1q by eCIRP would further contribute to propagation of the MerTK‐driven primary efferocytic dysfunction. Further investigation is needed to elucidate if MafB regulates MerTK directly or indirect mechanism involving C1q is possible. Taken together, these previous findings and our present data suggest that both MerTK and C1q suppression are likely to be involved in causing eCIRP‐induced microglial efferocytic dysfunction. Though C1q could be involved based on current findings, considering diverse roles of C1q in ischemic stroke settings, further detailed studies will be needed, which are beyond the scope of this project.

In the difficult landscape of IS drug development, precisely targeted therapeutic agents are a promising avenue. Immunosuppressive corticosteroid therapy is not recommended for IS, due to increased risk of opportunistic infections in the setting of post‐stroke immunodepression [57, 58]. Therefore, targeting specific pathways that promote tissue restoration and dampen exaggerated neuroinflammation are necessary. We demonstrated that C23 abrogates miR‐155 induction and enhances microglial efferocytosis, improving the stroke outcome measurements of infarct volume, and sensorimotor function. Recently, another group has reported that C23 improved blood‐brain‐barrier integrity in mice subjected to tMCAO [14]. Furthermore, a modified C23‐derived peptide significantly reduced infarct volume and sensorimotor deficits in a rhesus macaque IS model [13]. Furthermore, eCIRP/TLR signaling has been shown to promote NETosis of neutrophils [14, 16], and stimulate production and release of pro‐inflammatory cytokines in microglia and peripheral macrophages [12, 14], which are harmful downstream pathways in IS. eCIRP also signals through TREM‐1 in macrophages and endothelial cells to promote inflammation [9, 16]. Moreover, eCIRP damages neurons via inducing apoptosis [12] as well as IL‐6Rα/STAT3/Cdk‐mediated tau hyperphosphorylation [10]. Interestingly, C23 can also inhibit eCIRP/IL‐6Rα interaction albeit at much higher concentrations than needed for eCIRP/TLR4 inhibition [10]. Therefore, the beneficial effects of C23 in IS are probably due to inhibition of multiple deleterious mechanisms of eCIRP's role in ischemic stroke. Taken together, the present and recently published data conclude that C23 is a potentially efficacious peptide therapeutic for IS.

There are several limitations to our study. First, CIRP^−/−^ mice do not express the intracellular form of CIRP. Intracellular CIRP is known to be neuroprotective; however, in our extensive experience investigating eCIRP, we have observed that eCIRP's pro‐inflammatory effect is significantly more potent than any possible anti‐inflammatory effect conferred by intracellular CIRP [16, 59]. Efferocytosis in ischemic stroke is closely linked with neuronal death and occurs in a temporal pattern after tMCAO, beginning within hours, peaking around day 5 and continuing through later phases for days and may last for several weeks, depending on stroke severity. In addition, its dynamics involve both brain resident microglia as well as infiltrating peripheral macrophages with their actions overlapping in the later phases [60]. Thus, understanding the dynamics of efferocytosis in ischemic stroke is crucial for developing strategies for its regulation. The published literature widely supports that efferocytosis is a protective mechanism, but conversely, detrimental effects of excessive phagocytosis by microglia such as phagocytosis of “stressed‐but‐viable” neurons and excessive synaptic pruning have also been documented [60]. While our methodological approach specifically measures microglial efferocytosis in acute phase of ischemic stroke, the potential deleterious enhancement of microglial phagocytosis of live stressed neurons in this phase or synapses in the later phases could be an unfavorable consequence and would need to be further evaluated in future studies. It is also important to recognize that eCIRP has been shown to initiate multiple deleterious mechanisms that may interact with efferocytosis [14, 15, 16]. eCIRP might also affect rates of neuronal death or overall number of microglia independently, which may confound the interpretation of efferocytosis results. We also recognize that our in vitro studies utilized primary mouse microglia derived from mixed‐sex neonatal litters, while our in vivo stroke studies only used male mice. Since, sex differences exist in short‐ and long‐term stroke outcomes, along with sex‐specific differences in microglial response to stroke [61], it is critical to include female mice in future studies to dissect any sex‐specificity in eCIRP‐induced dysregulation of microglial efferocytosis in stroke. Our in vitro efferocytosis data utilized additional strategies and techniques, such as microscopic imaging with differential staining and flowcytometry with differential fluorescent labeling to control for such confounding factors. While our in vivo efferocytosis image acquisition strategies focused on the peri‐infarct region controlled for these factors, further validation is needed using more advanced whole brain imaging in future studies. Several mechanisms are known to regulate efferocytosis including a variety of players such as CD300A, CD5L, STAT6, TREM2, TAM receptors, milk fat globule EGF factor 8, peroxisome proliferator‐activated receptor γ, histone deacetylase 3 (HDAC3), sigma receptor‐1 and Rho/Rac1 [60]. We have shown that eCIRP downregulates Rac1 in macrophages via STAT3‐βPIX complex formation [36]. Furthermore, CD5L promotes autophagy‐mediated induction of ID3 to facilitate macrophage efferocytosis [62]. Interestingly, MafB has been shown to regulate CD5L in macrophages to promote atherosclerosis [63]. Notably, eCIRP via TLR4 has been shown to induce activation of HDAC3 in fibroblast‐like synoviocytes in the context of rheumatoid arthritis [64]. Moreover, HDAC3 inhibits macrophage efferocytosis and its deletion was shown to protect against ischemia‐induced retinopathy [65]. This supports the possibility that the eCIRP‐induced efferocytosis mechanism demonstrated in our study could also interact with other known regulators of efferocytosis as well.

Several limitations to the translational impact of our study necessitate further investigation in preclinical trials. First, it is mostly limited to the 24 h acute phase, which was selected primarily to facilitate the mechanistic study of eCIRP‐mediated acute‐phase microglial efferocytic dysfunction prior to significant influx of peripheral macrophages [66]. Our study showed that CIRP‐deficiency resulted in significant attenuation of brain infarction, local inflammation, neurological deficit, and mortality rate in 3‐day post tMCAO mice as well, suggesting that the detrimental role played by eCIRP in inducing microglial dysfunction extends beyond 24 h. Of note, one‐time administration of C23 immediately after wound closure not only improved microglial efferocytosis but also reduced the acute neurological deficits after stroke measured as Bederson score, which correlated with infarct volume along with decreased neuroinflammation in the acute phase. However, whether inhibiting eCIRP/TLR4 activation by C23 provides long‐term neuroprotection lasting in later phases of ischemic stroke remains to be determined. Given the high mortality rate observed with the vehicle over extended periods in the 60‐min tMCAO procedure used for the study, mice number in the cohort for the 7‐day survival supplementary data in this study were strictly minimized. Consequently, the 7‐day survival supplementary data primarily serve to highlight the limitations of long‐term survival in this specific tMCAO model configuration. Despite the high mortality in vehicle tMCAO limiting further continuation of long‐term outcome studies without modifying the current model, the limited C23 data suggests potential benefit of targeting eCIRP in acute ischemic stroke outcomes.

Although our preclinical C23 study does not simulate realistic clinical scenario, we would like to emphasize that we utilized C23 more as a tool for mechanistic study of eCIRP‐mediated efferocytic dysfunction, and less as a therapeutic candidate in the context of ischemic stroke at the current stage of investigation. This warrants further evaluation and validation in future studies with delayed C23 administration and tMCAO model modifications included to allow using longer experimental endpoints and a more sensitive and rigorous battery of sensorimotor and neurological behavior tests to fully evaluate the clinical relevance and efficacy of C23 in improving long‐term IS outcomes. Furthermore, the pharmacokinetic and pharmacodynamic properties of C23 have not yet been determined. Comprehensive assessment of C23's biodistribution, especially the degree of BBB penetration, is important not only to develop C23 for IS treatment, but also to further our understanding of C23's neuroprotective mechanism of action. Without direct evidence of C23 crossing the BBB, there is a possibility that C23 relies on BBB disruption for CNS access or that its effect could be primarily peripheral. However, our data clearly showed the beneficial effect of C23 in improving the microglial efferocytosis in ischemic stroke brain and potential improvement in 7‐day survival. Finally, our experiments only used young male mice, therefore further translational studies including female, aged, and hypertensive or diabetic mice are needed to evaluate C23 as an IS therapeutic.

In conclusion, we have demonstrated that eCIRP is a critical regulator of microglial efferocytosis via the TLR4/miR‐155/MafB pathway in IS. Our study shows promising improvements in IS outcome measurements, demonstrating the potential utility of C23 as a novel pharmacologic therapy.

## 4 Experimental Section

### 4.1 Experimental Animals

For experiments utilizing adult mice, 8 to 12‐wk‐old male wild‐type (WT) C57BL/6 mice were purchased from Charles River Laboratories (Fairfield, NJ), and age‐ and weight‐matched house‐bred CIRP^−/−^ mice were used. CIRP^−/−^ mice with C57BL/6 background were kindly provided by Dr. Jun Fujita (Kyoto Univesity, Kyoto, Japan) [67]. Mice were allowed to acclimate for 1 week before use in experiments. Mice were housed at the Center for Comparative Physiology at the Feinstein Institutes for Medical Research and in a temperature‐controlled room under 12 h light/12 h dark cycles and were fed standard chow diet ad libitum. For primary microglia isolation, breeding triads were closely monitored to precisely record the pups date of birth. House‐bred C57BL/6 and TLR4 deficient (TLR4^−/−^) neonatal mouse pups aged 0–3 days (P0‐P3) were utilized for primary microglia isolation. TLR4^−/−^ mice on the C57BL/6 background were obtained from Dr. Kevin Tracey (The Feinstein Institutes for Medical Research, Manhasset, NY). All experimental procedures were performed in accordance with the National Institutes of Health Guidelines for the Care and Use of Laboratory Animals. This study was approved by the Institutional Animal Care and Use Committee of the Feinstein Institute for Medical Research.

### 4.2 Temporary Middle Cerebral Artery Occlusion (tMCAO) Model of Ischemic Stroke

Male C57BL/6 and CIRP^−/−^ mice were utilized for tMCAO. tMCAO as previously described per the Longa method, adapted to mice with modifications [68]. Mice were weighed, and anesthesia was induced with 4% isoflurane, and maintained at 1.5%–2%. Body temperature was maintained at 37°C with a heating pad throughout the procedure. Mice were placed supine, the anterior neck fur was shaved, and residual fur removed with hair removal cream. The area was disinfected, and through a 1 cm midline incision the tissues were dissected to expose the left carotid bifurcation. The vagus nerve was identified, and care was taken to avoid injury to the structure. The proximal common carotid artery (CCA) was ligated, the distal external carotid artery (ECA) was ligated tightly, and the internal carotid artery (ICA) was ligated. A proximal ECA arteriotomy was performed, and a silicone‐tipped occluding suture (Doccol) was introduced and secured with a second tie on the ECA. The ICA was untied, and the occluding suture was advanced 8–10 mm to the origin of the middle cerebral artery (MCA) and fixed with silk ties on the ECA and ICA. The neck was closed with silk suture, and anesthesia withdrawn to allow for 1 h of awake ischemic time. The animal was anesthetized, and skin prepped in the same fashion, the occluding suture was slowly withdrawn, the ECA ligated proximally, and the CCA was untied to allow for reperfusion. Hemostasis was confirmed and cervical wound closed. Mice were randomly assigned to receive vehicle (Phosphate buffered Saline, PBS) or C23 (GenScript, Piscataway, NJ). Immediately after wound closure, mice were retro‐orbitally injected once with 0.1 mL vehicle or C23 (8 µg/g b.w.) dissolved in PBS. Sham mice underwent the same surgical procedures, but without intraluminal suture insertion. To ensure the reproducibility of experiments, all short‐term experiments were conducted at least three times. Animals were sacrificed 24 h or 3 days after reperfusion, and tissues were collected. Survival studies for 3‐day and 7‐day post‐tMCAO surgeries were also conducted. For survival studies, animals were monitered twice daily and administered 5 mg kg^−1^ body weight meloxicam via subcutaneous injection of 100 µL saline once daily. All mice received a single subcutaneous dose of 0.1 mg kg^−1^ buprenex immediately after tMCAO surgery.

### 4.3 CSF Collection and CIRP Enzyme‐linked Immunosorbent Assay (ELISA)

CSF was collected from mice 24 h after tMCAO or sham surgery, as described previously [69]. Briefly, mice were anesthetized with 4% isoflurane, shaved and placed in a stereotaxic frame (David Kopf Instruments), with gas anesthesia nosepiece attachment. A 1 cm long incision was made from the back of the head to the nape; muscles were retracted to expose the cisterna magna membrane with care to avoid injury and bleeding. Using a micromanipulator, a pulled glass capillary was used to carefully puncture the membrane overlying the cisterna magna and CSF was allowed to flow into the capillary for 5 min. At the end of CSF collection, mice were sacrificed. CSF was carefully inspected for blood contamination during collection and prior to transfer into sterile tubes, only pure CSF was flash frozen in liquid nitrogen, stored at −80°C, and used for experiments. Due to varying volumes of CSF collected, sample volumes were recorded immediately prior to beginning the ELISA protocol. Mouse CIRP ELISA kit (FineTest, catalog no. EM6504), was used for quantifying CSF eCIRP concentration as per manufacturer's protocol.

### 4.4 ECIRP and Peptide C23

Recombinant mouse CIRP (referred to as eCIRP) was prepared in‐house, with quality control measures performed as previously described [8]. C23 (GRGFSRGGGDRGYGG) was synthesized by GenScript USA Inc. (Piscataway, NJ), purified to > 95% by HPLC, and provided as a lyophilized powder. The peptide was resuspended in sterile PBS at desired concentration prior to treatment of cells or mice.

### 4.5 Stereotactic Hippocampal Injection

Male C57BL/6 mice were used for hippocampal injection and subjected to dorsal intra‐hippocampal injection per previously published protocol [70]. Briefly, anesthesia was induced with 4% isoflurane and maintained at 1%–2%. The scalp was shaved, residual fur removed with hair removal cream, the area was disinfected, and the animal was placed in the stereotactic apparatus (David Kopf Instruments). A 1 cm scalp incision was made and Bregma identified at the intersection of the bregmatic and sagittal sutures. Using a stereotactically mounted Dremel, a small burr hole was drilled in the skull at AP −2.0 mm, ML, 1.5 mm. 2 µL of eCIRP or PBS was loaded into microliter syringe (Hamilton) fitted with 33‐gauge needle (Hamilton). 1.17 µL (1 µg) of eCIRP was injected using the Quintessential Stereotaxic Injector (Stoelting) at a rate of 0.2 µL per min at coordinates AP −2.0 mm, ML, +1.5 mm, DL −1.5 mm. 5 min after completion of the injection, the syringe was withdrawn, hemostasis was confirmed, and the skin was closed with silk suture. Animals were sacrificed 24 h post‐operatively via cervical dislocation, and both ipsilateral and contralateral hippocampal tissues were collected and frozen in liquid nitrogen before storage at −80°C.

### 4.6 Bederson Score for Sensorimotor Deficit

Mice were scored 24 h after tMCAO using a 0–5 point modified version of the originally published Bederson score system as described previously [71]. No observable deficit is scored 0, forelimb flexion on tail raise is scored 1, decreased resistance to lateral push in addition to forelimb flexion is awarded a score of 2, circling behavior is scored 3, circling and spinning or “log rolling” around the cranial‐caudal axis is scored 4, and lack of spontaneous movement is scored 5.

### 4.7 Triphenyltetrazolium Chloride (TTC) Staining

Mice were sacrificed 24 h after tMCAO, brains were removed and cut into 2 mm thick coronal sections using a stainless‐steel brain matrix (Stoelting). Sections were stained at room temperature for 20 min in a 2% TTC solution in PBS, and fixed in 4% Paraformaldehyde (PFA). Photographs were taken, and edema‐corrected infarct volume was determined in a blinded manner using NIH ImageJ using a previously published method for quantification and edema correction [72].

### 4.8 Tissue Immunofluorescence Staining and Terminal Deoxynucleotidyl Transferase dUTP Nick End Labeling (TUNEL) Labeling

24 h after tMCAO, mice were anesthetized deeply with 5% isoflurane and transcardially perfused with pre‐perfusion buffer containing 0.5% sodium nitrite, 0.9% sodium chloride, and 0.1% heparin, followed by fresh 4% PFA. Brains were harvested and post‐fixed overnight at room temperature in 4% PFA and transferred the next day to 30% sucrose solution in PBS for 1–3 days. Brains were sliced into 40 µM sections on a freezing microtome. Sections were stained free floating. For efferocytosis experiments, tissues were permeabilized with 0.5% Triton X‐100 in PBS at 80°C for 15 min [73]. For other immunofluorescence experiments, tissues were permeabilized using 0.2% Triton X‐100 in 3% BSA in PBS. Tissues were blocked in 3% BSA in PBS for 1 h. Primary antibody staining was carried out at 4°C overnight, using rabbit anti‐mouse Ionized Calcium‐binding Adapted Molecule 1 (Iba‐1) (1:500; Wako Chemical, catalog no. 019–19741), and mouse anti‐mouse Neuronal Nuclei (NeuN) (1:250; EMD Millipore, catalog no. MAB377). For MerTK quantification, primary antibody staining was conducted in the same manner using rabbit anti‐mouse MerTK (1:200; Invitrogen, catalog no. 14‐5751‐82) and goat anti‐mouse Iba‐1 (1:500; Novus Biologicals, catalog no. NB100‐1028). Sections were washed with 0.2% Tween‐20 PBS and subsequently incubated with secondary antibodies for 1 h at room temperature with Alexa Fluor 532 goat anti‐mouse (1:500; Invitrogen, catalog no. A11002) and Alexa Fluor 647 goat anti‐rabbit (1:500; Invitrogen, catalog no. A21244) for efferocytosis and Alexa Fluor 594 donkey anti‐rabbit (1:500; Invitrogen, catalog no. A21207) and Alexa Fluor 647 donkey anti‐goat (1:500; Invitrogen, catalog no. A212447) for MerTK quantification. For efferocytosis, TUNEL staining was conducted using In situ cell death detection kit, Fluoroscein (Roche Diagnostics, catalog no. 11684795910), adapted for free floating samples. Free floating sections were stained in microcentrifuge tubes, with a 1:9 mixture of Tdt enzyme to labeling solution at 37°C for 1 h. Tissues were then washed with 0.2% Tween 20 in PBS, stained with 4',6‐diamidino‐2‐phenylindole (DAPI), mounted onto gelatin subbed slides, and coverslipped in ProlonGold antifade mountant (Invitrogen, catalog no. P36934).

### 4.9 Confocal Microscopy and Image Analysis

Brain tissues were imaged on a Zeiss LSM880 scanning confocal microscope (Carl Zeiss Imaging, Germany). The infarct region was defined as strongly positive TUNEL staining with a loss of NeuN staining, and the peri‐infarct region was identified as a circumscribing area 100–150 µM around the infarct. Z‐stack images were obtained in the peri‐infarct cortex using a 63x objective for the full thickness of the tissue section. For each brain sample, 3 images of 2–3 different tissue sections were obtained and analyzed. Efferocytosis quantification was carried out in Imaris (Bitplane) software. Briefly, surfaces were created using Iba‐1 staining for microglia and NeuN/TUNEL double positive staining for dead/dying neurons. Efferocytosed neuron surfaces were quantified using volume overlap classifier and calculated by dividing the number of completely engulfed TUNEL^+^ neurons by the total number of dead/dying neurons in image. For quantification of MerTK expression NIH ImageJ was utilized. Z‐stack images were converted into maximum intensity projections, threshold function was used on the Iba‐1 channel to create microglia ROIs, and ROIs were applied to MerTK channel to measure integrated density of MerTK staining in each ROI. The mean integrated density of MerTK fluorescence per microglia was calculated for each image, and 3 images obtained for ≥3 tissue sections were averaged and recorded for each animal.

### 4.10 Single‐Cell RNA Sequencing Analysis

Single cell transcriptome dataset GSE227651 was processed, explored, and Z‐score violin plots were generated using Trailmaker (https://app.trailmaker.parsebiosciences.com/; Parse Biosciences, analysis completed on 5/12/25). Unfiltered count matrices were uploaded and filtered by minimum number of transcripts per cell; 33013 for the sham samples and 26694 for the 24 h (day 1) tMCAO samples. Dying and dead cells were filtered by removing barcodes with > 8.89% mitochondrial reads. Outliers in the distribution of number of genes versus transcripts were removed by fitting a linear regression model (*p* < 0.000092). Probable doublets were filtered using the scDblFinder method (threshold range: 0.46238‐0.46758). Overall filtering rates after processing were 21.4% and 19.8% for sham and 24 h tMCAO, respectively. Data from high‐quality cells were normalized, integrated, and subjected to principal‐component analysis in Harmony. The Louvain method was used for clustering, and Uniform Manifold Approximation and Projection embedding was applied to visualize the data. Microglia‐specific genes were used to identify microglia by comparing cells in each cluster to other cells via the presto package and Wilcoxon rank‐sum test. Violin or dot plots for *Mafb, MiR155hg*, and *Mertk* were generated in the “Plots and Tables” module.

### 4.11 Isolation and Seeding of Primary Microglia

Primary microglia were isolated from C57BL/6 or TLR4^−/−^ P0‐P3 neonatal mouse pups, adapted from previously described protocols [74]. Briefly, whole brains were harvested in Hank's balanced salt solution and dissociated with Neural Tissue Dissociation Kit (P) (Miltenyi Biotec, catalog no. 130‐092‐628) per the manufacturer's protocol. Mixed glial cells were suspended in complete microglial medium (Dulbecco's Modified Eagle Medium (DMEM) containing 10% Fetal bovine serum (FBS), 100 U/mL penicillin/streptomycin, and 2 mM L‐glutamine additionally supplemented with 0.5 ng/mL recombinant murine granulocyte‐macrophage colony‐stimulating factor (GM‐CSF) (R&D Biosystems) and seeded in T‐175 culture flasks. Thereafter, culture medium was changed twice weekly. Microglia were recovered from flasks at DIV 10–21 by manual shaking, centrifuged at 400 g for 10 min, and resuspended in complete DMEM without GM‐CSF prior to seeding in tissue culture plates and rested overnight for at least 16 h before treatment.

### 4.12 Cell Culture and Treatments

All cells used were maintained in a humidified atmosphere at 37°C, 5% CO_2_. Mouse neuroblastoma Neuro‐2a (N2a) cells were kindly provided by Dr. Phillipe Marambaud (Feinstein Institutes for Medical Research, Manhasset, NY) as described before [10]. N2a cells were cultured in 1:1 DMEM/Opti‐minimum essential medium (Opti‐MEM) supplemented with 10% FBS and 100 U/mL penicillin/streptomycin. N2a cells were detached with 0.25% trypsin‐EDTA and seeded for experiments and passaged 1–2 times weekly. Microglia seeding density varied depending on downstream assay. For gene expression via RT‐qPCR or protein expression via Western blot, microglia were seeded at a density of 3 × 10^5^ cells per well. For efferocytosis assays and MerTK expression flow cytometry experiments, microglia were seeded at a density of 1 × 10^5^ cells per well in Upcell place (Nunc). After resting overnight, media was exchanged to fresh DMEM containing PBS, eCIRP, C23, or eCIRP + C23, at doses specified in each experiment, and incubated for 20 h before lysis or use in flow cytometry.

### 4.13 In Vitro Efferocytosis and MerTK Flow Cytometry Assays

Microglia were treated with increasing doses of eCIRP (0.1, 1, and 2.5 µg/mL) for 20 h. Cell death was induced in N2a cells by exposure to 600 J/m^2^ 24 h prior to staining and co‐incubation with microglia. After 24 h, N2a cells were detached, counted, and stained with CellTrace Carboxy fluorescein succinimidyl ester (CFSE) (Invitrogen, catalog no. C34554) per manufacturer's instructions. N2a cells were counted, resuspended to appropriate density, and fed to microglia (3 × 10^5^ cells/well) at 1:3 ratio of microglia to N2a cells. After 1 h co‐incubation, efferocytosis was terminated by washing with PBS, and cells detached for 15 min in cold PBS containing 1% FBS. Cells were then stained with antibodies, FITC anti mouse/human CD‐11b (1:200; BioLegend, catalog no.101206) and Brilliant Violent anti‐mouse F4/80 (1:20; BioLegend catalog no. 123132) and measured using BD FACSymphony flow cytometer (BD Biosciences, San Jose, CA, USA). FCS files were exported and analyzed in FlowJo 10.9.0 (BD Biosciences). Microglia were defined as CD11b^+^ and F4/80^+^ population. Median fluorescence intensity (MFI) of CFSE in the total microglia population and percentage of efferocytic microglia (microglia exhibiting CSFE fluorescence above no‐N2a cell negative control threshold) were recorded.

### 4.14 RNA Isolation and Real Time Quantitative PCR (RT‐qPCR)

Total RNA was isolated from cells or whole brains with RNAspin Mini kit (Cytiva), and miRNA was isolated using miRNeasy micro kit (Qiagen). RNA concentration was measured via Nanodrop. Conventional cDNA synthesis was carried out with 300 ng–1 µg of pure RNA using murine leukemia virus reverse transcriptase (Thermo Fisher Scientific, Waltham, MA). Quantitative PCR for relative mRNA expression was performed using specific primers (Table 1) and SYBR green (Applied Biosystems catalog no. 4312704), with GAPDH as endogenous control. For quantification of relative miR‐155 expression, cDNA synthesis was carried out using miRCURY LNA RT Kit (Qiagen, cat no. 33940); real‐time PCR reaction was conducted with miRCURY LNA SYBR Green PCR Kit (Qiagen, catalog no. 339346) with specific primers for mmu‐miR‐155‐5p (Qiagen Geneglobe, catalog no. YP02119303) and endogenous control miRNA miR‐103a‐3p (Qiagen Geneglobe, catalog no. YP02119303). All real‐time PCR reactions were carried out using QuantStudio3 real‐time thermocycler (Applied Biosystems), and the ΔΔCt method was employed to assess relative expression levels of all targets.

**TABLE 1:** RT‐qPCR primers used in this study.

RefSeq Accession #	Target	Forward primer (5’‐3’)	Reverse primer (5’‐3’)
NM_010658.3	*Mafb*	TGAATTTGCTGGCACTGCTG	AAGCACCATGCGGTTCATACA
NM_008587.3	*Mertk*	AAGGTCCCCGTCTGTCCTAA	GCGGGGAGGGGATTACTTTG
NM_007572.2	*C1qα*	AGAGGGGAGCCAGGAGC	CTTTCACGCCCTTCAGTCCT
NM_001411845.1	*GAPDH*	CATCACTGCCACCCAGAAGACTG	ATGCCAGTGAGCTTCCCGTTCAG

### 4.15 Western Blotting

Cells or brain tissues were lysed in ice cold RIPA lysis buffer (10 mM Tris buffered Saline (TBS) pH 7.5, containing phenylmethylsulfonyl fluoride, Na‐orthovanadate, protease and phosphatase inhibitor cocktails (Thermo fisher Scientific). Tubes were centrifuged at 12 000 rpm for 20 min at 4°C and protein containing supernatant collected in fresh tubes. Protein concentration was measured via DC protein assay (Bio‐Rad, catalog no. 5000111). Equal amounts of protein extracts (15 µg/well for cell lysate, 50 µg/well for brain lysate) were loaded into NuPAGE 4%–12% Bis‐Tris gels (Invitrogen, Thermo Fisher Scientific), separated via electrophoresis, and wet‐transferred onto nitrocellulose membranes. Membranes were blocked in 0.1% casein in TBS for 1 h at room temperature and then incubated in blocking solution containing 0.1% Tween‐20 overnight at 4°C with primary antibodies, rabbit anti‐MafB (1:500; Bethyl labs, catalog no. A700‐046), rabbit anti‐mouse Phospho‐Akt (Ser) (1:1000; Cell Signaling Technology, catalog no. 9271), rabbit anti‐mouse Akt (1:1000; Cell Signaling Technology, catalog no. 4691), rabbit anti‐mouse Rac1 (1:500; Sigma‐Aldrich, catalog no. 05–839), rabbit anti‐mouse Phospho‐Cofilin (Ser3) (1:1000; Cell Signaling Technology, catalog no. 3313S), and rabbit anti‐mouse Cofilin (1:1000; Cell Signaling Technology, catalog no. 5175S). After washing and incubation with infrared dye‐labeled secondary antibodies, membranes were imaged via Odyssey Clx Imaging system with Image Studio 5.2 software (Li‐Cor Biosciences). After target band imaging, membranes were then incubated and bands imaged in the same manner to measure mouse anti‐mouse β‐actin antibody (1:10 000; Sigma‐Aldrich, catalog no. A5441) as endogenous loading control. Densitometric quantification was done using NIH ImageJ.

### 4.16 Brain Tissue ELISAs

Protein‐containing brain tissue lysates from sham and tMCAO mice were generated as described for Western blotting methods. Brain IL‐6 and TNFα levels were quantified using BD OptEIA Mouse IL‐6 ELISA set (BD Biosciences, catalog no. 555240) and BD OptEIA Mouse TNFα ELISA set (BD Biosciences, catalog no. 558534) per manufacturer's protocol. Data are normalized to total protein content measured in brain tissue lysate immediately prior to beginning the ELISA protocol.

### 4.17 Statistical Analysis

Data were analyzed using GraphPad Prism version 8.0 graphing and statistical analysis software (GraphPad Software Inc., La Jolla, CA) and presented as mean ± standard error of the mean (SEM). For two group comparisons, unpaired two‐tailed Student's t test was used. For multigroup comparisons One‐way analysis of variance (ANOVA) and post‐hoc Tukey's multiple comparisons test was employed. To analyze experimental data with two independent variables we used two‐way ANOVA or mixed‐effects model (for data with missing values) and Sidak's multiple comparisons test. All data were tested for normality by the Shapiro‐Wilk test. Survival rates were compared using Kaplan‐Meier survival plots and a log‐rank test. α was set at 0.05, thus comparisons with p < 0.05 were considered statistically significant differences.

## Author Contributions

DL performed surgical procedures, conducted experiments, analyzed and interpreted data, and drafted and revised the manuscript. DA conducted experiments and analyzed and interpreted data. AS supervised drafting and revising the manuscript. AS and PW conceived the idea and critically reviewed the manuscript. All authors read and approved the final manuscript.

## Funding

This work was supported by the National Institutes of Health (NIH) grant RF1 AG091371 (AS, PW), R01 AA028947 (PW), R35 GM118337 (PW), and T32 AI155392.

## Ethics Statement

Animal experiments were performed following the NIH Guide for the Care and Use of Laboratory Animals. All experiments were approved by the Feinstein Institutes for Medical Research Institutional Animal Care and Use Committee (IACUC; #23‐0531).

## Conflicts of Interest

The authors declare no conflicts of interest.

## Supporting information




**Supporting File**: advs77053‐sup‐0001‐SuppMat.docx.

## Data Availability

The data that support the findings of this study are available from the corresponding author upon reasonable request. The single‐cell RNA sequencing dataset analyzed in this study is publicly available under accession number GSE227651 in the NCBI Gene Expression Omnibus repository.
