# Supplementary material for: Extracellular CIRP Dysregulates Microglial Efferocytosis in Acute Ischemic Stroke via the TLR4/miR‐155/MafB Axis
Source: Adv Sci (Weinh). 2026 Aug 15;13(53):e77053. doi: 10.1002/advs.77053 (PMC13477243; doi:10.1002/advs.77053)
Supplement: Supplementary file 1 [file ADVS-13-e77053-s001.docx]

**Supplementary Data Figures**

**Supplementary Fig. 1.** Representative z-stack orthogonal projection of single microglia showing MerTK costaining with Iba-1 in the merged channel confocal image from a zoomed in region of the peri-infarct brain tissue of CIRP^-/-^ tMCAO mice, stained for nuclei (DAPI, blue), microglia (Iba-1, magenta), and MerTK (green). Magnification 630x, scale bar, 20 µM.

**Supplementary Fig. 2.** Violin plots showing expression Z-scores for **a** miR-155 host gene *MiR155hg*, and **b** *Mafb* in microglia cluster from sham and day 3 tMCAO from GSE227651. Dot plots showing relative gene expression of **c** *MiR155hg*, and **b** *Mafb* in microglia cluster from sham, day 1 tMCAO and day 3 tMCAO from GSE227651.

**Supplementary Fig. 3.** Immunofluorescent quantification of MafB expression in peri-infarct microglia of WT and CIRP^-/-^ tMCAO mice. **a** Representative confocal images of peri-infarct brain tissues in WT tMCAO and CIRP^-/-^ tMCAO mice, with staining for nuclei (DAPI, blue), microglia (Iba-1, magenta), and MafB (green). Magnification 400x, scale bar, 50 µM. **b** Quantified immunofluorescence intensity measurement of microglial MafB expression. (*n* = 5-6/group; mean ± SEM; * *p* = 0.004; Unpaired *t*-test).

**Supplementary Fig. 4.** eCIRP causes efferocytic dysfunction in primary microglia *in vitro*. Primary microglia were treated with PBS or eCIRP (1 µg/mL) for 20 h. Apoptotic N2a cells were labeled with Celltracker Red and NHS-Biotin, and added to microglia at a ratio of 1:3 microglia:N2a cells, co-incubated for 1 h and washed with PBS. Wells were stained with FITC-Streptavidin, to label only un-efferocytosed (extracellular compartment) N2a cells and fragments and fixed in 4% PFA. Representative confocal images are shown, with staining for nuclei (DAPI, blue), N2a cells (Celltracker Red red), and extracellular N2a cells (Streptavidin, green). 630x magnification, scale bar, 10 µM. Celltracker Red staining without green Streptavidin staining indicates engulfed N2a corpses, Celltracker Red with green Streptavidin staining indicates un-engulfed N2a corpses. Selected efferocytosis events are indicated with arrows, incomplete events are indicated with arrowheads.

**Supplementary Fig. 5.** Primary microglia downregulate MerTK mRNA expression *in vitro with eCIRP stimulation via TLR4*. **a** *Mertk* mRNA expression in primary microglia treated with PBS or eCIRP (1 µg/mL) for 20 h. (*n* = 11/group; mean ± SEM; * *p* < 0.001; Unpaired *t*-test). **b** *Mertk* mRNA expression in primary microglia isolated from WT or TLR4^-/-^ mice treated with PBS or eCIRP (1 µg/mL) for 20 h. (*n* = 6/group; mean ± SEM; * *p* = 0.0108 WT eCIRP vs. WT PBS, ns. *p* = 0.6620 TLR4^-/-^ eCIRP vs. TLR4^-/-^ PBS; two-way ANOVA, Sidak’s multiple comparisons test).

**Supplementary Fig. 6.** C1q expression in primary microglia is suppressed in response to eCIRP *in vitro*. Gene expression of *C1qα* in primary microglia treated with indicated doses of eCIRP for 20 h. (*n* = 5-6; * *p* < 0.0001 vs. 0 µg/mL eCIRP; one-way ANOVA, Tukey’s multiple comparisons test).

**Supplementary Fig. 7.** miR-155 mimic downregulated MafB expression in immortalized murine microglia. Representative Western blot and quantification of BV-2 cells (murine microglial cell line) treated with PBS or eCIRP (1 µg/mL) as well as cells transfected with a miR-155 mimic. (*n* = 5-6/group; * *p* = 0.0117 eCIRP vs. PBS; * *p* = 0.0002 miR-155 mimic vs. PBS; one-way ANOVA, Tukey’s multiple comparisons test).

**Supplementary Fig. 8.** WT mice were subjected to tMCAO surgery and received retroorbital injection of C23 or vehicle (PBS) and were monitored for 7 days for humane endpoints, and differences in survival were determined using Kaplan‐Meier survival plots and a log‐rank test. *n* = 4 mice/group. Hazard ratios (HR): vehicle tMCAO, 8.034; C23 tMCAO, 0.1245. **p* = 0.0169 versus vehicle.

**Supplementary Methods**

**Immunofluorescence microscopy and image processing:**

Immunofluorescence staining, image acquisition and processing were carried out as described in main text methods. Orthogonal views were generated via orthogonal view function in NIH ImageJ, with crosshair centered on area of enriched MerTK staining.

**Immunofluorescence microscopy for MafB expression**:

The tMCAO model, tissue processing and immunofluorescence staining, and analysis were conducted as described in the main text Methods. Antibody staining was carried out using the same protocol as MerTK staining. Tissues were stained with primary antibodies rabbit anti-MafB (1:200; Bethyl labs, catalog no. A700-046) and goat anti-mouse Iba-1 (1:500; Novus Biologicals, catalog no. NB100-1028), and secondary antibodies Alexa Fluor 488 donkey anti-rabbit (1:500; Invitrogen, catalog no. A21206). Peri-infarct regions of brain tissues were imaged on a Zeiss LSM880 scanning confocal microscope (Carl Zeiss Imaging, Germany) using a 40x objective. Image analysis was conducted using NIH ImageJ. Z-stack images were converted into maximum intensity projections, threshold function was used on the Iba-1 channel to create microglia ROIs, and ROIs were applied to MafB channel to measure integrated density of MafB staining in each ROI. Results are reported in arbitrary fluorescence units (AU).

***In vitro* efferocytosis imaging assay:**

Method was adapted from previously described protocol^1^, with modifications detailed below. Primary microglia were seeded onto poly-D-lysine pre-coated 8-well chamber slides (Mattek, catalog no. CCS-8) and treated with PBS or 1 µg/mL eCIRP for 20 h. Cell death was induced in N2a cells via exposure to 600 J/m^2^ UV irradiation 24 h prior to staining and co-incubation with microglia. N2a cells were stained with CellTracker Red CMTPX (Invitrogen, catalog no. C32552) per manufacturer’s protocol, and EZ-Link™ NHS-Biotin (Thermo Scientific, catalog no. 20217) prior to co-incubation. N2a cells were added to adherent microglia at a ratio of 1:3 (microglia effector: N2a prey cells) and incubated for 1 h, washed with PBS, and stained with FITC-streptavidin (Thermo Scientific, catalog no SA1001) and fixed in 4% PFA and stained with DAPI. Cells were imaged using LSM880 scanning confocal microscope (Carl Zeiss Imaging, Germany) with 63x objective. Qualitative image analysis was carried out in NIH ImageJ with internalized efferocytosis cargo defined as CellTracker Red positive and FITC-streptavidin negative.

**miR-155 mimic transfection of BV-2 cells:**

Mouse microglial cell line BV-2 cells were generously provided by Dr. Phillipe Marambaud (Feinstein Institutes for Medical Research, Manhasset, NY) as previously described^2^. BV-2 cells were cultured in the same manner described in main text methods, maintained in completed DMEM (without rmGM-CSF) and seeded for experiments at a density of 1 x 10^5^ cells/well overnight prior to transfection. BV-2 cells were left transfected with HiPerFect Transfection reagent (Qiagen, catalog no. 301704) mixed with mmu-miR-155-5p miRCURY LNA miRNA Mimic (Qiagen Geneglobe, catalog no. YM00470919-ADB) or left un-transfected for 24 h. After 24 h un-transfected cells were treated with PBS or 1 µg/mL eCIRP for 20 h. Cells were lysed for protein content and Western Blots for MafB and β-actin were carried out as described in main text methods.

**Supplementary References**

1. Taruc, K., Yin, C., Wootton, D. G. & Heit, B. Quantification of efferocytosis by single-cell fluorescence microscopy. *J. Vis. Exp.* (2018) doi:10.3791/58149.
2. Sharma, A., Brenner, M., Jacob, A., Marambaud, P. & Wang, P. Extracellular CIRP activates the IL-6Rα/STAT3/Cdk5 pathway in neurons. *Mol. Neurobiol.* **58**, 3628–3640 (2021).
